# Combustion Conditions are the Major Drivers of the Variability in Organic Aerosol Chemical Composition and Secondary Organic Aerosol Formation in Emissions from Simulated Wildland Fires

**DOI:** 10.1021/acsestair.6c00258

**Published:** 2026-09-15

**Authors:** Muhammad I. Abdurrahman, Michael T. Caraway, Nicholas Kusumo, Thomas Carroll, Kruthika V. Kumar, Anita Anosike, Ariana M. Deegan, Ryan P. Poland, John T. Allen, Mac A. Callaham, Joseph J. O’Brien, Edward C. Fortner, Amanda Frossard, Geoffrey D. Smith, Rawad Saleh

**Affiliations:** † School of Environmental, Civil, Agricultural, and Mechanical Engineering, 1355 University of Georgia, Athens, Georgia 30602, United States; ‡ Department of Physics and Astronomy, University of Georgia, Athens, Georgia 30602, United States; § Department of Chemistry, University of Georgia, Athens, Georgia 30602, United States; ∥ U.S. Department of Agriculture Forest Service, Athens Prescribed Fire Science Laboratory, Southern Research Station, Athens, Georgia 30602, United States; ⊥ 53777 Aerodyne Research Inc, Billerica, Massachusetts 01821, United States

**Keywords:** combustion efficiency, organic nitrogen, photochemical aging, brown carbon, particulate emissions

## Abstract

Wildland-fire emissions exhibit large variability in aerosol physicochemical properties and potential for forming secondary organic aerosol (SOA). Whether this variability is primarily driven by differences in fuel type or combustion conditions remains unresolved. To address this gap, we performed simulated wildland-fire experiments using fuel beds that contained either surface fuels only or a duff layer underneath the surface fuels. We varied the dry mass loading and moisture content of the surface fuels within ranges encountered in prescribed fires in the Southeastern U.S. Less efficient combustion, which resulted from increased dry mass loading, increased moisture content, or including duff in the fuel bed, produced less light-absorbing organic aerosol (OA) and formed more SOA. Including duff also produced OA with higher nitrogen content. A proof-of-concept experiment using surface fuels conditioned to reproduce inefficient combustion conditions similar to those of duff emitted OA with nitrogen content, light-absorption properties, and SOA formation potential comparable to those of duff OA emissions. Given the profound differences in molecular composition between surface fuels and duff, these results provide strong evidence that the difference in combustion conditions between duff and surface fuels was the major driver of the observed differences in OA physicochemical properties and SOA formation potential.

## Graphical abstract

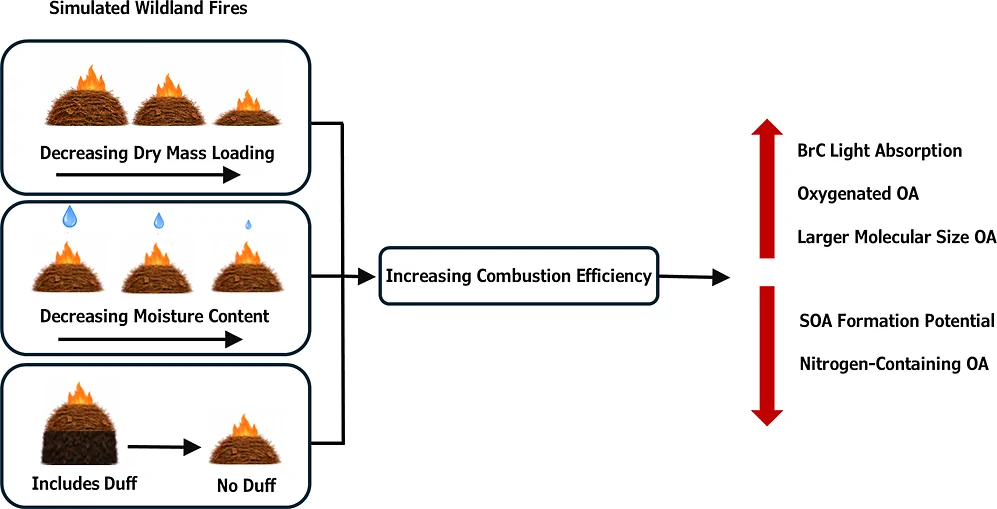

## 1 Introduction

Prescribed fires are an essential land-management tool for reducing the accumulation of hazardous fuels, thereby lowering the risk of catastrophic wildfires,^1,2^ as well as for promoting ecosystem health and maintaining biodiversity.^3,4^ Between 1985 and 2020, prescribed fires constituted an annual average of 11 million acres across the United States,^5^ with over 70% occurring in the Southeast.^6^ Prescribed fires are strategically conducted within a prescription window determined by a set of favorable environmental conditions that allow for achieving land-management goals in terms of fuel consumption, while maintaining controlled fire behavior and minimizing smoke exposure.^4,7,8^ A key factor in determining the prescription window is fuel moisture content. To ensure consumption of surface fuels (recently senesced forest litter that collects on the forest floor) that are typically targeted in prescribed fires, the fuels should be dry enough to ignite, but not so dry as to risk extreme fire behavior. In regions that contain duff (a layer of partially decomposed surface fuels situated between the surface fuels and mineral soil), prescribed fires are designed to avoid duff ignition, which is detrimental to plant health^9–11^ and can lead to prolonged smoldering and large amounts of smoke emissions.^12,13^ Avoiding duff ignition requires conducting prescribed fires such that surface fuels are available for combustion (dry enough), but duff is not (has high moisture content). This situation can be attainable within a few days after rainfall,^4,14^ leveraging the fact that due to the compactness of duff, adjustment of its moisture content to atmospheric conditions exhibits longer lag times compared to surface fuels. Though rare, duff can ignite unintentionally during prescribed fires, largely due to difficulties in accurately estimating its ignition threshold.^13,15^ The likelihood of duff ignition increases substantially when dry conditions persist for long periods of time, as is the case in drought-induced wildfires.^12,16,17^

A major barrier to the widespread implementation of prescribed fires, both from a public and regulatory perspective, is concern over the effect of smoke emissions on air quality.^18,19^ Therefore, there is a need to understand how smoke emissions and their impacts vary within the parameters that define the prescription window to inform the safe and effective use of prescribed fires. However, a persistent challenge in quantifying the impacts of smoke emissions from prescribed fires, and wildland fires in general, lies in the large variability in the reported physicochemical properties of the smoke aerosol^20–22^ as well as the potential for forming secondary organic aerosol (SOA).^23,24^ While most laboratory aging experiments of emissions from combustion of wildland fuels reported a net increase in organic aerosol (OA) concentration due to simulated atmospheric aging,^23,25–29^ field campaigns observed inconsistent behavior with outcomes ranging from substantial net SOA production to net OA mass loss.^23^ Moreover, the chemical composition of the smoke aerosol and its transformation during atmospheric processing vary markedly from fire to fire.^23,30^ The formation pathways and atmospheric transformation of species such as nitrogen-containing OA molecules^31^ and non-methane organic vapors (which are SOA precursors) remain poorly constrained.^21,30^

Additionally, there is a large uncertainty in the effect of smoke emissions from wildland fires on atmospheric radiative balance, driven in part by limited constraints on the light-absorption properties of light-absorbing OA (brown carbon; BrC).^32,33^ Although BrC absorbs light less efficiently than black carbon (BC), it is emitted in much higher mass concentrations in wildland fires, making it a potentially significant contributor to the absorption of solar radiation in the atmosphere.^33–35^ Accounting for BrC absorption has shown the potential for improving the closure between chemical transport model calculations and remote-sensing observations of atmospheric absorption attributed to aerosol emissions from wildland fires.^36^ However, accurate representation of BrC absorption in models is still hindered by the large variability in BrC light-absorption properties reported in the literature.^33,37–40^

There is evidence that the large variability in the physicochemical properties of OA and SOA formation potential of smoke emissions in wildland fires is in part determined by the variability in combustion conditions,^20,21,29,41–46^ which emerge from the complex interactions between fuel-bed characteristics and environmental conditions.^29,47^ In this study, we build on this evidence and conduct simulated wildland fires in the laboratory to explore how the variability in fuel-bed characteristics and environmental conditions within the prescription window in Southeastern prescribed fires impacts OA chemical composition, SOA formation potential, and BrC light-absorption properties.

## 2 Methods

### 2.1 Experimental Design

This study was part of the second Georgia Wildland-fire Simulation Experiment (G-WISE-2), an intensive laboratory campaign conducted in April-May 2025 at the Athens Prescribed Fire Science Laboratory of the U.S. Forest Service’s Southern Research Station, located on the campus of the University of Georgia. The major goal of G-WISE-2 was to investigate how the variability in combustion conditions within the prescription window modulates smoke production and physicochemical properties of the resulting smoke emissions in prescribed fires, building on insights gained from the first campaign (G-WISE-1), conducted in October-November 2022.^47^

In G-WISE-1, we investigated the effects of differences in (1) dry fuel composition, by constructing fuel beds representative of three ecoregions in the Southeastern U.S. (Blue Ridge, Piedmont, Coastal Plain), and (2) moisture content, by conditioning the fuels to two levels of moisture content representative of either the mid-point of the prescription window or drought-induced wildfires. Both aerosol optical properties^44,45^ and SOA formation potential^29^ were found to be determined by the combustion conditions that emerged from the dry fuel-bed composition and moisture content.

In G-WISE-2, we constructed fuel beds representative of one ecoregion (Coastal Plain) and designed an experimental matrix to investigate the effects of (1) mass loading of surface fuels encountered during prescribed fires, (2) moisture content of the surface fuels at the edges of the prescription window, (3) presence of duff in the fuel bed, (4) duff moisture content, and (5) wind speed. One of the major findings from G-WISE-1 was the profound differences in smoke production and physicochemical properties between fuel beds that involved duff ignition and those that did not,^29,41,42,44–46^ which we attributed to differences in combustion conditions. Specifically, due to its compactness^48,49^ and lower carbon content^48,50,51^ compared to surface fuels, duff combusts significantly less efficiently than the surface fuels.^29,44^ However, the G-WISE-1 experiments did not rule out the possibility that the differences in smoke emissions between duff and surface fuels were due to differences in fuel composition (fuel type) rather than combustion conditions. To address this question, in G-WISE-2, we designed a proof-of-concept fuel bed, dubbed “faux duff” (FD), that included surface fuels only but was conditioned to replicate similar inefficient combustion conditions to those of duff. To that end, the fuel bed had a high mass loading, high moisture content, and was compacted to limit oxygen availability and lower the combustion temperature, similar to duff. We note that, unlike the other fuel beds, which were designed to simulate realistic fuel beds as observed in the field (Section 2.2), the compactness (3D structure) of FD is not representative of field conditions, and its high moisture content is outside the prescription window.

The analyses reported in this paper were performed on a subset of the experimental permutations (Table 1) in G-WISE-2. In all experimental permutations considered here, wind speed was held constant at 1 m s^–1^. The baseline experimental permutation (SF1) included surface fuels only and featured mid-range dry mass loading (750 g/m^2^) and moisture content (12% for fine fuels and 30%–43% for woody fuels). The effect of dry mass loading was investigated by holding the baseline moisture content constant and varying dry mass loading to lower (SF2; 250 g/m^2^) and upper (SF3; 1500 g/m^2^) limits, based on field observations of prescribed fires in the Southeast.^52,53^ The effect of moisture content was investigated by holding the baseline dry mass loading constant (750 g/m^2^) and varying the moisture content to lower (SF4; 5% for fine fuels and 1%–2.5% for woody fuels) and upper (SF5; 18% for fine fuels and 30%–43% for woody fuels) limits for the prescription window in the Southeast.^4,14^ The effect of duff ignition was investigated by holding the baseline dry mass loading and moisture content of the surface fuels constant, and including a duff layer underneath the surface fuels (D1). There is no set range of prescription moisture content for duff. In practice, prescribed fires in regions that contain duff are typically conducted a few days after rainfall^4,14^ to avoid duff ignition, leveraging the fact that the adjustment of moisture content of duff to atmospheric conditions exhibits longer lag times compared to surface fuels. In rare cases, the moisture content of duff can be below the ignition threshold in prescribed fires, leading to unintentional duff combustion. Because of the large uncertainty in the ignition threshold of duff,^13,15^ the moisture content of the duff layer in D1 was 5%, which was lower than what would be expected in prescribed fires,^4,14^ but was chosen to ensure its ignition in the experiments. As described above, the FD experimental permutation was not representative of realistic field conditions but was designed to achieve combustion conditions similar to those of duff, but using surface fuels only. Representative photographs of SF, D1, and FD fuel beds are given in Figure S1 of the Supporting Information.

**Table 1:** Subset of the G-WISE-2 Experimental Matrix Discussed in this Paper (Full Experimental Matrix is given in Table S1)

experimental permutation	dry mass loading		moisture content		
	surface fuels^a^ (g/m^2^)	duff (g/m^2^)	fine	woody	duff
SF1 (baseline)	750	0	12%	30%–43%	N/A
SF2	250	0	12%	30%–43%	N/A
SF3	1500	0	12%	30%–43%	N/A
SF4	750	0	5%	1%–2.5%	N/A
SF5	750	0	18%	30%–43%	N/A
D1	750	5844	12%	30%–43%	5%
FD	1500	0	30%	30%–43%	N/A

a The dry mass composition of surface fuels is 52% pine needles, 24% other fine fuels, and 24% woody fuels.

### 2.2 Preparation of Fuel Beds and Combustion Experiments

Fuels were collected from Fort Stewart Army Base, located in the Southern Coastal Plain region of Georgia, United States, from a unit that had undergone more than 15 years of fire exclusion, and were used to construct fuel beds that replicated the composition and 3D structure typical of fuel beds in this region.^52–54^ The fuels were categorized into surface fuels and duff. The surface fuels were gently raked or clipped above ground and were then classified into fine fuels (pine needles and a mix of grasses, sedges, vines, and forbs) and woody fuels of varying diameters. The woody fuels were further classified into 1 h, 10 h, and 100 h fuels, based on the characteristic time scales over which their moisture content equilibrates with ambient humidity.^55^ The duff was extracted as square blocks (10 cm × 10 cm × 10 cm) using a stainless-steel cutting tool. Sand and other soil material adhering to the underside of the duff were removed using a soil knife. Collected samples were placed in sealed plastic zip-lock bags and stored at room temperature in the laboratory.

The fuels were oven-dried to a moisture content of approximately 1%, then conditioned to the target moisture content in an enclosed humidification chamber equipped with an industrial ultrasonic humidifier (Global Industrial, Model #246135). The moisture content was periodically monitored using a Moisture & Solids Analyzer (Computrac, Model MAX 4000XL) until the target moisture content was reached. Once conditioned, fuels were sorted and sealed in zip-lock bags for storage prior to the experiments. Representative samples of pine needles, grasses, and duff were subsampled and analyzed for carbon content and nitrogen content using an elemental analyzer-isotope ratio mass spectrometer (EA-IRMS).

The fuel beds were constructed with an area of 0.5 m^2^, consistent with the concept of a “wildland fuel cell” unit, beyond which fire behavior becomes spatially independent,^8^ in order to capture the small-scale interdependence between fire behavior and smoke production encountered in field conditions. The dry mass distribution of the surface fuels was approximately 52% pine needles, 24% other fine fuels, and 24% woody fuels. For the fuel bed with a duff layer (D1), the duff blocks were tightly arranged beneath the surface fuels across the entire fuel-bed area. For this fuel bed, the duff and surface fuels constituted approximately 86% and 14% of the total dry mass, respectively. Compaction of the FD fuel bed was achieved by laboratory personnel standing on a wooden board (i.e., using body weight) placed on top of the surface fuels for 2 min prior to the burn.

The combustion experiments were conducted in a 990 m^3^ burn room equipped with an array of fans to simulate wind and promote rapid mixing of emissions. Sampling lines were extended from the burn room to an adjacent instrument room for online measurements and collection of filter samples for offline analyses. Fuel beds were placed on a digital scale to monitor real-time fuel consumption during each burn. For SF1-SF5, combustion typically concluded within approximately 10 min, as indicated by observation of real-time video recording. In contrast, D1 and FD exhibited smoldering combustion for up to 2 h. Following the conclusion of the combustion process, the smoke in the burn room reached well-mixed conditions within approximately 10 min, as inferred from stable CO concentration measured with a CO analyzer (N300, Teledyne).

In all experiments, online measurements and filter sampling began once well-mixed conditions were established. For online measurements, the smoke was diluted by approximately a factor of 16 using a Dekati diluter (DI-1000). The G-WISE-2 instrumentation and data analysis relevant to this study are described in the subsequent sections.

### 2.3 Quantification and Chemical Speciation of Smoke Aerosol

A Soot Particle Aerosol Mass Spectrometer (SP-AMS; Aerodyne Research Inc.) was deployed to measure the chemical composition and mass concentration of the smoke aerosol. The SP-AMS is a modified version of the high-resolution time-of-flight aerosol mass spectrometer (HR-ToF-AMS) that, in addition to nonrefractory organic materials, enables quantitative detection of refractory BC (rBC) and associated refractory organic material.^56^ The SP-AMS was operated by alternating between laser-OFF and laser-ON modes. In laser-OFF mode, the SP-AMS operates analogously to a conventional AMS, relying on a resistively heated tungsten vaporizer maintained at 600 °C to vaporize non-refractory aerosol components.^56^ Under this configuration, refractory components are not efficiently vaporized and therefore remain largely undetected. In contrast, laser-ON mode employs both the tungsten vaporizer and a 1064 nm intracavity laser. Laser absorption by strongly light-absorbing particles heats them to sublimation or incandescence temperatures, thereby efficiently vaporizing them. The resulting vapors are then ionized by 70 eV electron impact and analyzed using high-resolution time-of-flight mass spectrometry.^56^

The analysis presented in this paper is based on the laser-ON configuration of the SP-AMS. Compared to the laser-OFF mode, the laser-ON configuration consistently yielded higher OA concentrations, averaging 37% greater mass concentrations across all experimental permutations. Despite this difference in OA levels, the mass spectra obtained from the two configurations were comparable, indicating no significant difference in OA chemical composition. This suggests that the discrepancy in measured OA concentrations stemmed from differences in collection efficiency related to particle bounce of the vaporizer rather than from differences in the types of OA fragments detected.^56^ It can also be due to reduced ionization efficiency in the laser-OFF mode, since particles vaporized by the laser are generated at the intersection of the electron beam and the ion extraction axis of the mass spectrometer, whereas those vaporized by the vaporizer are generated away from the ion extraction axis.^56^

### 2.4 Photochemical Aging

Emissions were photochemically aged in an Aerodyne Potential Aerosol Mass oxidation flow reactor (PAM OFR). The OFR comprises a 13 L cylindrical flow chamber equipped with low-pressure mercury lamps that emit UV radiation at 185 and 254 nm, thereby enabling in situ generation of OH radicals via the photolysis of O_2_ and H_2_O at λ = 185 nm and O_3_ at λ = 254 nm.^57^ OH exposure in the OFR depends on flow rate (residence time), UV light intensity, water vapor concentration, and external OH reactivity, defined as the rate at which reactive species within the OFR scavenge OH radicals.^28,58,59^ Using the OFR internal calibration based on the parametrization of Peng et al.^59^ as a starting point, we targeted an OH exposure of approximately 7.9 × 10^11^ molecules cm^–3^ s, which is equivalent to 6 days of atmospheric aging, assuming a daily average ambient OH concentration of 1.5 × 10^6^ molecules cm^–3^.^60,61^ The flow rate was maintained at 10 SLPM, resulting in a calculated mean residence time of approximately 1.3 min. To account for variations in ambient temperature and water vapor concentration across experiments, the UV lamp intensity was adjusted at the start of each experiment to achieve the target OH exposure.

The actual OH exposures were estimated from the consumption of toluene, assuming pseudo-first-order reaction kinetics.^62–64^ Toluene concentrations were measured using an AROMA-VOC analyzer (model #ARVOC-181-RSO) at a time resolution of 14 min. OH exposures estimated from toluene consumption in the OFR (Supporting Information Section S1) ranged from 5.46 × 10^11^ molecules cm^–3^ s to 7.26 × 10^11^ molecules cm^–3^ s (approximately 4–5 days of equivalent atmospheric aging) (Supporting Information Table S2). The difference in OH exposures obtained from the estimation equation and toluene consumption is likely due to uncertainty in assumed external OH reactivity of the smoke constituents. A detailed description of the OH exposure estimation procedure is provided in the Supporting Information (Section S1).

We calculated the change in OA concentration (OA enhancement) from the difference between the concentrations of aged and fresh OA^25,28,29^ by alternating the OFR operation between the UV lights-OFF and UV lights-ON modes. Details of this procedure are provided in the Supporting Information (Section S2). OA enhancement values greater than 0% indicate a net increase in OA mass concentration due to SOA formation. It should be noted that the extent of SOA formation can potentially be larger than indicated by OA enhancement. The reason is that the oxidation of some semi-volatile organic compounds (SVOCs) can lead to their repartitioning from the particle phase back into the gas phase, thereby reducing OA concentrations and potentially masking the full extent of SOA formation.^65^

### 2.5 Combustion Conditions

We characterized combustion conditions using two complementary metrics: the modified combustion efficiency (MCE) and BC/OA from measurements of emitted gases and particles. MCE is a widely used metric for quantifying the combustion efficiency of various combustion systems, including biomass burning, and is defined as

1 $$MCE=\frac{\Delta {CO}_{2}}{\Delta {CO}_{2}+\Delta CO}$$

where, ΔCO_2_ and ΔCO represent mixing ratios of CO_2_ and CO above background levels.^66,67^ The mixing ratios of CO_2_ and CO were measured at a 1 min time resolution using a CO_2_ analyzer (N360M, Teledyne) and a CO analyzer (N300, Teledyne), respectively. High MCE values (approaching unity) indicate efficient combustion characterized by higher temperatures, whereas low MCE values indicate oxygen-deprived combustion characterized by low temperatures.^66,68,69^ In the combustion of wildland fuels, MCE values above 0.9 are commonly associated with flaming-dominated combustion, whereas values below 0.9 indicate smoldering-dominated combustion.^20,69,70^

BC/OA has likewise been proposed as a proxy for combustion conditions, with higher values associated with more efficient combustion.^71–73^ We calculated BC/OA using two different methods: (1) online measurements using the SP-AMS and (2) offline measurements using a thermal-optical organic-carbon elemental-carbon (OCEC) analyzer (Sunset Laboratory, model 5 L) following the NIOSH-870 protocol.^74^ For the OCEC thermal-optical analysis, quartz (Q) and quartz-behind-Teflon (QBT) filters were collected and analyzed. EC was quantified directly from the Q filter, while OC was corrected for gas-phase adsorption by subtracting the OC measured on the QBT filter from that measured on the Q filter. We assumed BC is equivalent to EC and converted OC to OA using a factor of 1.8, consistent with the OA/OC ratios retrieved from the SP-AMS and with our prior work.^75^

### 2.6 Retrieval of Light-Absorption Properties

The wavelength-dependent imaginary part of the refractive index (*k*) of BrC aerosol was retrieved using an optical-closure approach, which involved fitting aerosol absorption coefficients (*b*_abs_) obtained from Mie calculations to those measured with a photoacoustic spectrometer (Multi-PAS III)^76^ at three wavelengths (420, 532, and 780 nm), with *k* as a free parameter.^44,73,77,78^ The spectral dependence of *k* was quantified using a power-law relationship^73^

2 $$k_{\lambda }=k_{550}{\left(\frac{\lambda }{550}\right)}^{-w}$$

where *k*_λ_ is the imaginary part of the refractive index at wavelength λ, *k*_550_ is the *k* value at 550 nm, and *w* characterizes the wavelength dependence. Thus, the wavelength-dependent *k* can be expressed using the two parameters, *k*_550_ and *w*.

The contribution of BC to the total aerosol absorption was accounted for in the optical-closure analysis assuming that the BC and OA (i.e., BrC) particles were externally mixed. The absorption attributable to BrC was then obtained by subtracting the contribution of BC from the total measured absorption

3 $$b_{abs,BrC}=b_{abs}-b_{abs,BC}$$

where, *b*_abs_ is the total measured absorption coefficient and *b*_abs,BC_ is the BC absorption coefficient calculated using Mie theory. We employed a BC complex refractive index of *m* = 1.85 + 1.71*i* ^79^ and assumed that the BC size distribution has the same shape as the total aerosol size distribution measured by a scanning mobility particle sizer (SMPS), but scaled by the BC/OA. We performed the Mie calculations assuming homogeneous spherical BrC particles with a real part of the refractive index of 1.55.^79^ We note that the assumed spherical particle shape and external mixing do not capture the true morphology and mixing state,^80,81^ and can affect the retrieved light-absorption properties.^82,83^ However, Saleh et al.^82^ demonstrated that for use in climate and chemical transport models, the particle morphology and mixing-state assumptions used in retrievals of BrC light absorption properties should be consistent with those adopted in the intended models. Most regional and global climate and chemical transport models calculate aerosol optical properties using Mie theory, which assumes spherical particles, and have employed both internal-mixing^84–86^ and external-mixing^36,87–89^ assumptions. Therefore, we advise using an external-mixing assumption if the light-absorption properties obtained from this study are to be employed in radiative-transfer calculations.

## 3 Results and Discussion

### 3.1 Variability in Combustion Conditions

Figure 1a depicts the variability in combustion conditions across the experimental permutations, quantified using the MCE and the BC/OA derived from the OCEC analyzer. Figure 1b compares BC/OA derived from the SP-AMS to that derived from the OCEC analyzer. BC/OA from the two instruments was in good agreement for SF1-SF5, which had relatively high MCE (0.90–0.94). However, for D1 and FD, both characterized by smoldering-dominated combustion (low MCE, 0.78 and 0.79, respectively), the SP-AMS yielded significantly higher BC/OA than the OCEC analyzer. This discrepancy is likely due to the charring (i.e., pyrolytic carbonization) of OA in the SP-AMS induced by the 1064 nm laser, which artificially inflated the rBC signals. Similar behavior was observed previously using a Single-Particle Soot Photometer (SP2), which also uses a 1064 nm intracavity laser.^90^

**Figure 1:**
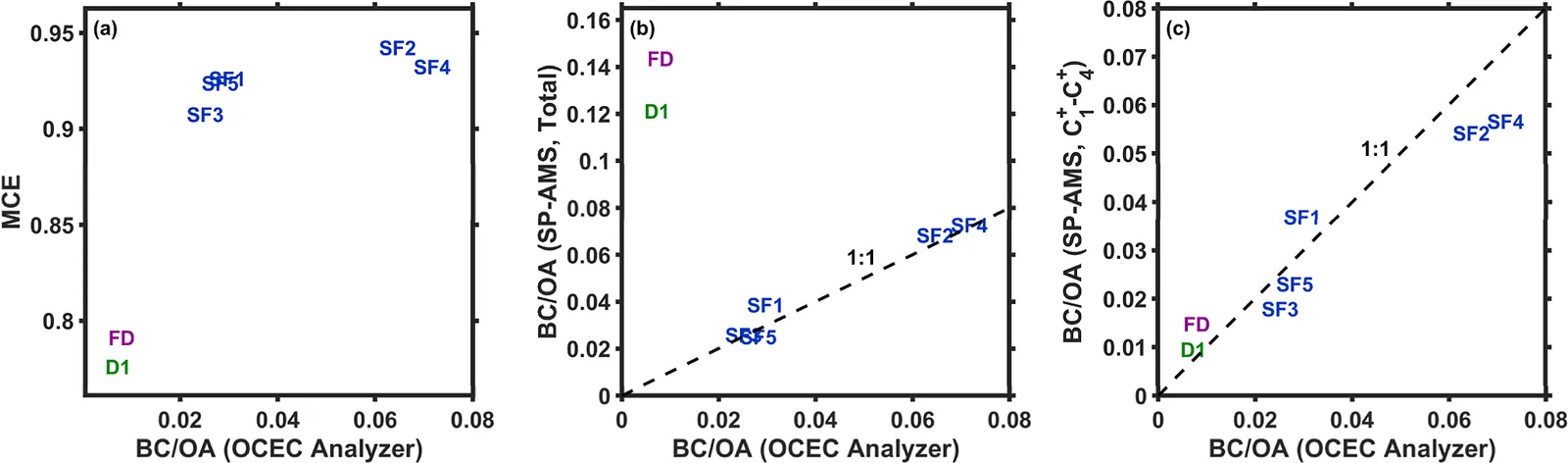
Ratio of black carbon to organic aerosol (BC/OA) retrieved from the OCEC analyzer plotted against (a) modified combustion efficiency (MCE), (b) BC/OA retrieved from the SP-AMS integrated over all *m/z* values, and (c) BC/OA retrieved from the SP-AMS integrated over C_1_^+^–C_4_^+^ (*m/z* 12 to *m/z* 48). Each point corresponds to an experimental permutation labeled by its identifier, as described in Table 1: SF1 (baseline; surface fuels only), SF2/SF3 (low/high dry mass loading; surface fuels only), SF4/SF5 (low/high moisture content; surface fuels only), D1 (duff + surface fuels), and FD (faux duff; surface fuels only; high mass loading; very high moisture content; compacted). Standard deviations are smaller than marker size. Numerical values of measurements (means and standard deviations) are reported in Supporting Information Table S3. Details on measurement uncertainty are provided in Supporting Information Section S3.

Examination of the SP-AMS rBC mass spectra (Supporting Information Figure S2) provides further insight into this interpretation. For SF1-SF5, the rBC spectra were dominated by low-carbon-number cluster ions (C_1_^+^–C_4_^+^, *m/z* 12 to *m/z* 48), which accounted for 70%–96% of the total rBC signal, in agreement with previous biomass-burning studies.^91,92^ In contrast, D1 and FD exhibited greater contributions (92% and 90%, respectively) from higher-carbon-number cluster ions (C_5_^+^–C_25_^+^, *m/z* 60 to *m/z* 300), with the largest contribution coming from C_5_^+^. As shown in Figure 1c, when the rBC mass in the SP-AMS was calculated using only the low-carbon-number cluster ions across all experimental permutations, we observed good agreement with the OCEC-derived BC/OA for all experimental permutations, including D1 and FD. The enrichment of the larger carbon fragments in D1 and FD implies that the charring artifact is likely associated with the generation of C_5_^+^–C_25_^+^ in the SP-AMS, whereas, in agreement with previous studies,^91,92^ C_1_^+^–C_4_^+^ appear to better represent the BC signal from combustion emissions.

We speculate that the reason the OA in D1 and FD was more susceptible to charring compared to the OA in SF1-SF5 is that it was generated at substantially lower combustion temperatures, leaving a higher fraction of the OC-to-BC conversion (i.e., soot formation^93–95^ ) incomplete. Consequently, the high temperatures induced by absorption of the 1064 nm laser pushed these incomplete transformations over the BC-formation threshold. Due to this bias, regardless of the reason, we use BC/OA from the OCEC analyzer as a metric for combustion conditions in the subsequent sections.

MCE and BC/OA paint a similar qualitative picture of the effect of variability in fuel-bed characteristics (composition and dry mass loading) and environmental conditions (manifested as moisture content) on combustion conditions. Across experimental permutations involving combustion of surface fuels only, increasing dry mass loading (SF2 < SF1 < SF3) led to more oxygen-deprived, lower-temperature combustion, which manifested as a decrease in both MCE and BC/OA. A similar trend was observed with increasing moisture content (SF4 < SF1 < SF5). With higher moisture content, more of the thermal energy released from the combustion process was diverted to water evaporation, leading to lower-temperature combustion, which manifested as a decrease in both MCE and BC/OA.

Including duff in the fuel bed had a more profound impact on combustion conditions than the variations in mass loading and moisture content of the surface fuels, with D1 exhibiting substantially lower MCE and BC/OA compared to SF1-SF5. This observation is in agreement with previous studies reporting that duff combustion occurs at lower temperatures compared to surface fuels.^12,13,29,44^ The reason is that the relatively higher bulk density^16,48^ and lower carbon content^48,50,51^ of duff relative to surface fuels led to more oxygen deprivation, thus less efficient combustion. This interpretation is reinforced by the fact that FD exhibited combustion conditions closely resembling those of D1, yielding comparable MCE and BC/OA, despite the compositional differences between the two fuel beds. As described in Section 2.1, FD is used in subsequent sections as a proof-of-concept experimental permutation to test whether reproducing combustion conditions similar to those of duff using surface fuels only would yield smoke with physicochemical properties similar to those of the smoke emitted from duff combustion. This comparison will enable us to determine whether the distinctive physicochemical properties of duff smoke^29,44,45^ arise primarily from combustion conditions or from fuel composition (fuel type).

While BC/OA and MCE exhibited similar qualitative trends with varying combustion conditions, the comparison between the utility of the two metrics warrants further elaboration. As evident in Figure 1a, MCE is effective at resolving the variability between smoldering-dominated combustion (D1 and FD) and more flaming combustion (SF1-SF5), but saturates as it approaches unity (i.e., CO approaches zero) at higher efficiency, limiting its utility to resolve the variability at these conditions. BC/OA behaves in the opposite way. Because of the high sensitivity of OC-to-BC conversion (soot formation) to temperature,^93–95^ BC/OA is effective at resolving the variability in combustion conditions at the relatively high-efficiency part of the spectrum, where combustion temperatures are expected to be high. This is consistent with the observation of Bruns et al.,^26^ who reported up to an order-of-magnitude variability in BC/OA over a narrow MCE range between 0.94 and 0.97. However, BC/OA is less effective at resolving variability in the smoldering-dominated part of the spectrum, as BC/OA approaches zero.

### 3.2 Secondary Organic Aerosol Formation

OA enhancement due to SOA formation for the different experimental permutations is plotted in Figure 2 as a function of BC/OA. For experimental permutations involving combustion of surface fuels only, less efficient combustion, driven by an increase in dry mass loading or moisture content, was associated with larger OA enhancement. As described in Section 3.1, this is because an increase in both dry mass loading and moisture content of the surface fuels hindered the completeness of the combustion process (conversion of carbon to CO_2_) and produced smoke with higher levels of incomplete-combustion products, including SOA precursors.^21,29^

**Figure 2:**
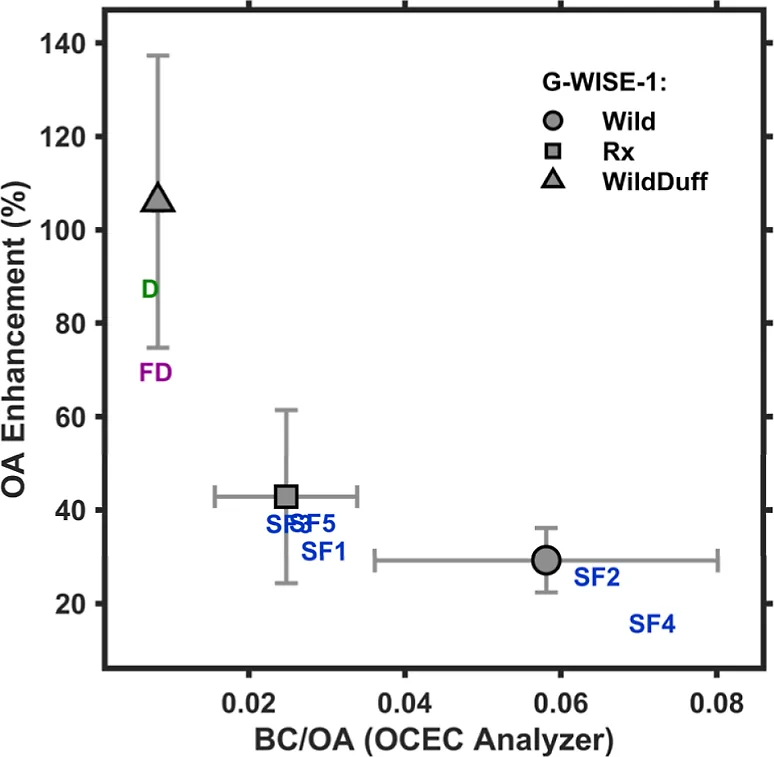
Dependence of organic aerosol (OA) enhancement on combustion conditions across all experimental permutations as a function of ratio of black carbon to organic aerosol (BC/OA) retrieved using the OCEC analyzer. Each point corresponds to an experimental permutation labeled by its identifier, as described in Table 1: SF1 (baseline; surface fuels only), SF2/SF3 (low/high dry mass loading; surface fuels only), SF4/SF5 (low/high moisture content; surface fuels only), D1 (duff + surface fuels), and FD (faux duff; surface fuels only; high mass loading; very high moisture content; compacted). Standard deviations are smaller than marker size. Numerical values of measurements (means and standard deviations) are reported in Supporting Information Table S3. Details on measurement uncertainty are provided in Section 3 (S3) of the Supporting Information. OA enhancement values from the G-WISE-1 are included for comparison.

D1 exhibited the least efficient combustion and the largest OA enhancement. Moreover, FD, which had the same dry composition as SF1-SF5 but comparable combustion conditions to D1 (Figure 1), had a large OA enhancement, close to that of D1. This finding indicates that the higher SOA production in D1 compared to SF1-SF5 is driven by differences in combustion conditions rather than fuel composition.

Also shown in Figure 2 is the OA enhancement reported from G-WISE-1, which examined smoke emissions from fuels collected from three ecoregions in the Southeastern U.S. (Piedmont, Coastal Plain, and Blue Ridge) at moisture contents representative of prescribed fires and drought-induced wildfires. The Piedmont and Coastal Plain fuel beds contained surface fuels only. The Blue Ridge fuel bed contained duff and surface fuels, but the duff was only available for combustion under the wildfire conditions - it was too moist to ignite under prescribed-fire conditions. The G-WISE-1 OA-enhancement clustered into three groups based on the combustion conditions: (1) drought-induced wildfires including surface fuels only (wild), (2) prescribed fires (Rx), and (3) drought-induced wildfires including duff and surface fuels (WildDuff). Wild, which had the most efficient combustion among the three groups, as shown by its high BC/OA, exhibited the lowest average OA enhancement. Rx had less efficient combustion than Wild and exhibited a larger average OA enhancement. WildDuff had the least efficient combustion, characterized by the lowest BC/OA, and exhibited the largest average OA enhancement. These results are consistent with those from this study and indicate that the observed variability in SOA formation potential is predominantly driven by combustion conditions, even when comparing fuels from different ecoregions.

During biomass combustion, SOA precursors are produced through multiple overlapping processes. Some are emitted directly from the combustion process as intermediate (incomplete-combustion) products, while others are released from the biomass matrix due to the elevated temperatures associated with the release of thermal energy from the combustion process, which triggers distillation and chemical transformations such as depolymerization and fragmentation.^96,97^ The compositional profile of these species is expected to depend on fuel composition. For example, the combustion of surface fuels releases significantly more terpenes than duff,^98^ which have been depleted during the transformation (decomposition) of surface fuels to duff. These different species have varying SOA formation potentials. However, the results in Figure 2 indicate that the effect of combustion conditions on the aggregate production of SOA precursors, thus SOA formation potential, is more important than differences in fuel composition. Furthermore, our results provide evidence that variability in combustion conditions (captured via MCE and BC/OA) can explain, at least in part, the large variability in SOA formation potential of biomass-burning emissions reported in previous studies.^23,24^

### 3.3 Chemical Composition of Organic Aerosol

#### 3.3.1 Relative Abundance of Chemical Families

We categorized the SP-AMS high-resolution spectral ions into five chemical families based on their elemental composition: C_*x*_H_*y*_^+^ (reduced organic fragment ions; with no oxygen or nitrogen), C_*x*_H_*y*_O^+^ (oxidized organic fragment ions with a single oxygen atom), C_*x*_H_*y*_O_>1_^+^ (oxidized organic fragment ions with two or more oxygen atoms), C_*x*_H_*y*_N^+^ (reduced nitrogen-containing organic fragment ions), and C_*x*_H_*y*_ON^+^ (oxidized nitrogen-containing organic fragment ions). The fresh OA mass spectra of the different experimental permutations are shown in Figures 3a–c and S3a–e. Across experimental permutations involving combustion of surface fuels only (SF1-SF5), variations in dry mass loading or moisture content did not lead to significant differences in the mass spectra or relative abundance of chemical families. The spectra were dominated by C_*x*_H_*y*_^+^, C_*x*_H_*y*_O^+^, and C_*x*_H_*y*_O_>1_^+^, with only small variations in the relative abundance of each family.

**Figure 3:**
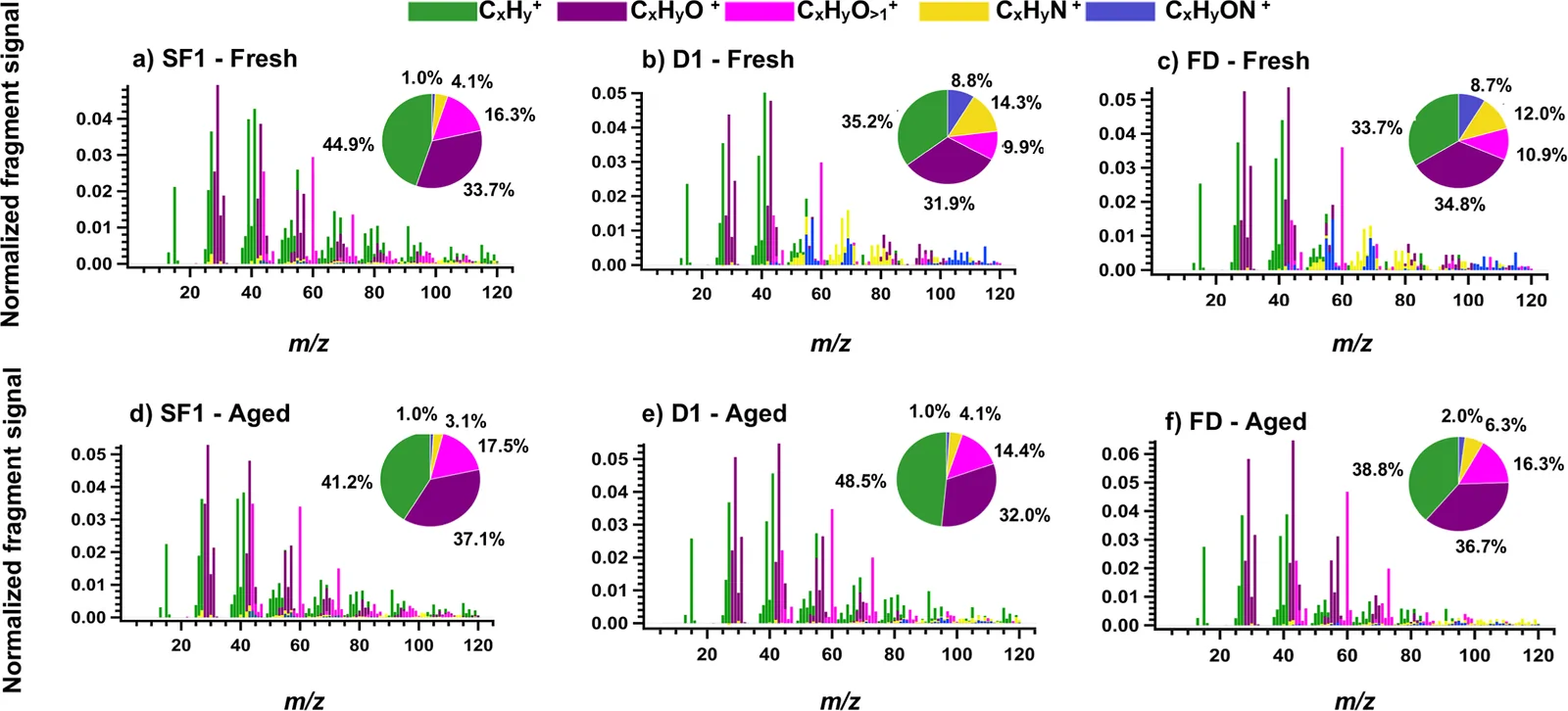
Normalized high-resolution mass spectra of organic fragment ions and relative abundance of chemical families in the organic aerosol (OA) measured by the SP-AMS for select experimental permutations (SF1, D1, and FD) for (a–c) fresh emissions and (d–f) aged emissions. Plots for the other experimental permutations are given in Supporting Information Figure S3.

Including duff in the fuel bed led to notable differences in OA chemical composition compared to fuel beds that included surface fuels only. A major distinguishing feature is the enhanced contribution of nitrogen-containing organic fragment ions (C_*x*_H_*y*_N^+^ and C_*x*_H_*y*_ON^+^) in D1 compared to SF1. In G-WISE-1, we observed qualitatively similar results using electrospray ionization Fourier-transform ion cyclotron resonance mass spectrometry (ESI-FTICR-MS), which we attributed to differences in fuel composition.^44^ Specifically, the decomposition of organic matter (surface fuels) during duff formation involves microbial consumption of carbon (released as CO_2_) as well as translocation of nitrogen (mainly through fungal hyphal networks) into the duff material, leading to a lower carbon-to-nitrogen mass ratio (C/N) in duff compared to surface fuels.^49,99–103^ Indeed, the duff and surface fuels in this study had C/N of 45 and 140, respectively. Despite this large difference in C/N between duff and surface fuels, FD produced OA with mass spectra that closely resembled those of D1, indicating that the observed enhancement in nitrogen-containing organic species in D1 compared to SF1-SF5 was driven by differences in combustion conditions rather than by fuel composition.

This finding is consistent with that of Chen et al.,^50^ who showed that low-temperature smoldering combustion of biomass favored the emission of nitrogen-containing organic compounds. It is worth mentioning that these nitrogen-containing organic compounds are likely dominated by non-aromatic species emitted as distillation products, i.e., molecules that volatilize directly from the fuel rather than from the combustion process.^50^ This is not the case for nitroaromatics, however, which are formed directly by the combustion process and whose emissions are favored under high-temperature combustion.^104,105^

Across experimental permutations involving surface fuels only (SF1-SF5), photochemical aging produced similar changes in OA chemical composition, consistently showing a decrease in the fraction of C_*x*_H_*y*_^+^ and a corresponding increase in the fractions of C_*x*_H_*y*_O^+^ and C_*x*_H_*y*_O_>1_^+^ (Figures 3d and S3f–j), and an overall increase in O/C (Figure S4). This can be attributed to the OH-induced oxidation of fresh OA and the formation of SOA in the OFR, and is in general agreement with the findings from previous studies that investigated the aging of biomass-burning OA.^23,25,106,107^

Photochemical aging of D1 (Figure 3e) and FD (Figure 3f) emissions tells a different story, demonstrating an increase in the fractions of C_*x*_H_*y*_^+^, C_*x*_H_*y*_O^+^, and C_*x*_H_*y*_O^+^_>1_, along with a marked decrease in the fractions of C_*x*_H_*y*_N^+^ and C_*x*_H_*y*_ON^+^. The increase in the fractions of C_*x*_H_*y*_O^+^ and C_*x*_H_*y*_O_>1_^+^ with photochemical aging is expected and can also be explained by OH-driven oxidation, as in the case of SF1-SF5. The increase in the fractions of C_*x*_H_*y*_^+^ and the simultaneous decrease in those of C_*x*_H_*y*_N^+^ and C_*x*_H_*y*_ON^+^ in D1 and FD can be explained as follows. Photochemical aging can cause fragmentation of nitrogen-containing organics, in which cleavage of functional groups removes nitrogen, leaving smaller, likely reduced, organic molecules.^108^ Overall, the results in Figure 3 illustrate that the chemical composition and aging behavior of FD were different from those of SF1-SF5 and closely resembled D1, indicating that the role of combustion conditions in determining OA chemistry was more prominent than that of fuel composition.

#### 3.3.2 SP-AMS Characteristic Markers

SP-AMS characteristic markers, including *f*_44_, *f*_43_, and *f*_60_, provide insights into the source and oxidation level of OA.^109–111^ *f*_44_ is the organic signal at *m/z* 44 (mostly CO_2_^+^) relative to the total organics signal and is generally associated with oxygenated functional groups such as carboxylic acids.^110,112^ While *f*_44_ has been primarily used as a tracer for aged OA and SOA, it has also been shown to constitute significant components of fresh biomass-burning OA.^51,109,113^ *f*_43_ (mostly C_3_H_7_^+^ and C_2_H_3_O^+^) reflects contributions from more reduced, hydrocarbon-like organic species.^110,111^ *f*_60_ (mostly C_2_H_4_O_2_^+^) is commonly used as a biomass-burning tracer due to its association with levoglucosan and related anhydrous sugars produced during cellulose pyrolysis in biomass combustion.^110^ Studies have shown that *f*_73_ (predominantly C_3_H_5_O_2_^+^) can also be used as a biomass-burning tracer,^110,114–116^ and as shown in the Supporting Information (Figure S5), *f*_73_ exhibited good agreement with *f*_60_ across all experimental permutations, indicating that the trends and interpretations presented herein for *f*_60_ could similarly be described using *f*_73_.

Figure 4a depicts *f*_44_ versus *f*_43_ for fresh and aged OA. For fresh OA, experimental permutations involving surface fuels only (SF1-SF5) showed that more inefficient combustion, associated with increasing dry mass loading or moisture content, resulted in OA with lower *f*_44_ and higher *f*_43_. There was a deviation in SF3, however, which exhibited lower *f*_43_ than SF1 despite having lower combustion efficiency. While the reason for the slight deviation in SF3 is unclear, the overall trend of *f*_44_ and *f*_43_ suggests that higher combustion temperatures and greater oxygen availability promote the formation of more oxygenated OA.

**Figure 4:**
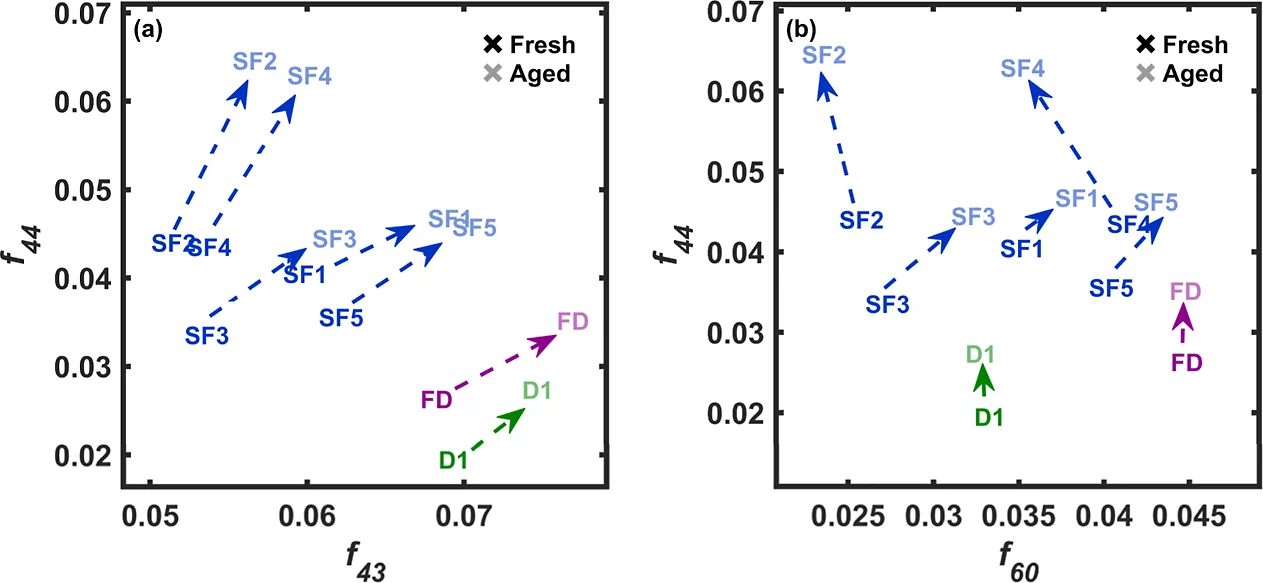
SP-AMS characteristic markers of organic aerosol (OA) presented in (a) *f*_44_ versus *f*_43_ space and (b) *f*_44_ versus *f*_60_ space. Each point corresponds to an experimental permutation labeled by its identifier, as described in Table 1: SF1 (baseline; surface fuels only), SF2/SF3 (low/high dry mass loading; surface fuels only), SF4/SF5 (low/high moisture content; surface fuels only), D1 (duff + surface fuels), and FD (faux duff; surface fuels only; high mass loading; very high moisture content; compacted). Bold identifiers denote fresh OA and faded identifiers denote aged OA, with arrows indicating the direction of photochemical aging. Standard deviations are smaller than the marker size. Numerical values of measurements (means and standard deviations) are reported in Table S4. Details on measurement uncertainty are provided in Supporting Information Section S3.

D1 followed the same trend. Having had the least efficient combustion, it also exhibited the lowest *f*_44_ and the highest *f*_43_ (Figure 4a). FD yielded *f*_44_ and *f*_43_ values that closely followed those of D1, further supporting the interpretation that this trend was driven by combustion conditions rather than fuel composition. This observation qualitatively agrees with field measurements^117^ and laboratory experiments^51,106^ that reported formation of more oxidized OA with more efficient combustion. As expected and widely reported by previous studies,^23,25,110,111^ OA became more oxidized due to photochemical aging, leading to an increase in *f*_44_ and *f*_43_ across all experimental permutations (Figure 4a).

As shown in Figure 4b, all experimental permutations produced appreciable *f*_60_ signals, exceeding the 0.3% threshold reported for ambient OA impacted by biomass-burning emissions.^110^ As opposed to the OA characteristics discussed thus far (SOA formation potential, organic-nitrogen content, *f*_44_, and *f*_43_) which reflect contributions from a broad range of combustion products, *f*_60_ is dominated by levoglucosan and related anhydrous sugars and, therefore, depends strongly on the abundance of their precursor, cellulose, in the fuel.^118,119^ This renders the dependence of *f*_60_ on combustion conditions more nuanced. Focusing first on SF1-SF5, the fuel beds included fine and woody fuels (Table 1). The woody fuels have higher cellulose content than fine fuels.^120–123^ Due to the low moisture content, the consumption of woody fuels in SF4 was substantially higher than the other experimental permutations involving surface fuels only (Table S5), suggesting that the elevated *f*_60_ was associated with emissions from the woody fuels. Combustion conditions still play an important role, as demonstrated by the large *f*_60_ in FD. Despite consumption of fine fuels only (negligible woody fuel consumption), FD had the highest *f*_60_ among all experimental permutations, suggesting that inefficient smoldering combustion favors cellulose pyrolysis and, consequently, enhanced levoglucosan emissions.

D1 exhibited mid-range *f*_60_ emissions compared to the other experimental permutations, which can be attributed to the interplay between fuel composition and combustion conditions. On the one hand, duff is expected to have lower content of levoglucosan precursors compared to surface fuels due to the partial decomposition of cellulose during duff formation.^49,121,124,125^ On the other hand, the less efficient combustion of duff is conducive for forming levoglucosan from the existing precursors.

As shown in Figure 4b, photochemical aging produced mixed effects on *f*_60_. On the one hand, the two experimental permutations that had the most efficient combustion (SF2 and SF4) exhibited a decrease in *f*_60_ with aging, consistent with previous reports that aging of OA can lead to photochemical degradation of levoglucosan,^23,25^ as well as SOA formation diluting the *f*_60_ signal by adding OA molecules at other *m/z*. On the other hand, the rest of the experimental permutations (SF1, SF3, SF5, D1, and FD) exhibited either stable or slightly elevated *f*_60_ after aging. There is evidence that biomass-burning SOA has fragments that contribute to *f*_60_.^25,110^ It is possible that this effect was prominent in these experimental permutations because of the relatively large amount of SOA (Figure 2). These results suggest that while *f*_60_ remains a useful marker of fresh biomass-burning emissions, its utility could degrade with smoke age. Both the aging-induced loss of levoglucosan and the formation of oxygenated secondary species can alter *f*_60_, thereby limiting its reliability as a quantitative marker in aged biomass-burning smoke.

### 3.4 Light-Absorption Properties of the Organic Aerosol

The light-absorption properties of OA from all experimental permutations are presented in Figure 5 on the log_10_(*k*_550_)-*w* framework proposed by Saleh.^33^ Within this framework, larger *k*_550_ and smaller *w* correspond to progressively darker (more-absorbing) BrC, allowing optical classification of BrC into very weakly absorbing (VW-BrC), weakly absorbing (W-BrC), moderately absorbing (M-BrC), and strongly absorbing (S-BrC). As described in Section 2.6, *k*_550_ and *w* were retrieved assuming externally mixed BC and OA. The extent of internal versus external mixing is expected to vary across experiments, with more internal mixing with decreasing BC/OA. We have previously shown that assuming external versus internal mixing with core–shell morphology led to less than 30% and 40% differences in *k*_550_ and *w*, respectively, for a similar range in BC/OA as in this study.^73^ Consequently, while applying the same external-mixing assumption across experiments could affect the absolute values of *k*_550_ and *w*, it is not expected to alter the observed trend in Figure 5.

**Figure 5:**
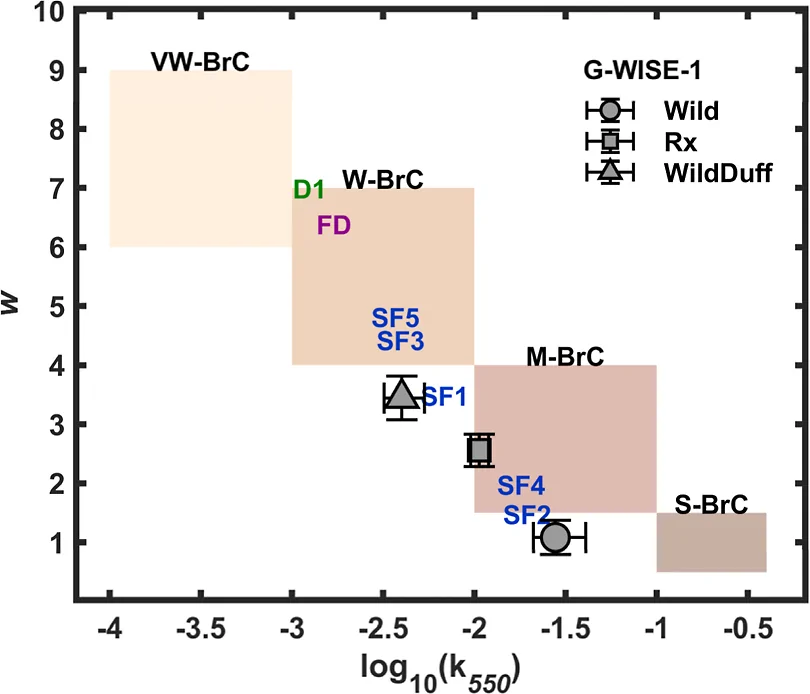
Light-absorption properties of organic aerosol (OA) presented on the log_10_(*k*_550_)-*w* framework for the different experimental permutations in this study and those reported in Glenn et al.^44^ Shaded regions denote the classification of light-absorbing OA (brown carbon; BrC) into very weakly absorbing (VW-BrC), weakly absorbing (W-BrC), moderately absorbing (M-BrC), and strongly absorbing (S-BrC).^33^ Each point corresponds to an experimental permutation labeled by its identifier, as described in Table 1: SF1 (baseline; surface fuels only), SF2/SF3 (low/high dry mass loading; surface fuels only), SF4/SF5 (low/high moisture content; surface fuels only), D1 (duff + surface fuels), and FD (faux duff; surface fuels only; high mass loading; very high moisture content; compacted). Standard deviations for data in this study are smaller than the marker size. Numerical values of measurements (means and standard deviations) are reported in Table S6. Details on measurement uncertainty are provided in Supporting Information Section S3 (S3). *k*_550_ and *w* values from the G-WISE-1 (Glenn et al.)^44^ are included for comparison.

For SF1-SF5, less efficient combustion, driven by higher dry mass loading or moisture content, emitted less-absorbing BrC, as evidenced by smaller *k*_550_ and larger *w*. Furthermore, D1 and FD, which exhibited less efficient combustion than SF1-SF5, emitted less-absorbing BrC (smaller *k*_550_ and larger *w*). Consistent with the findings in the previous sections regarding OA chemical composition and SOA formation potential, the fact that the BrC emissions of FD more closely resembled those of D1 than SF1-SF5 indicates that the role of combustion conditions in determining BrC light-absorption properties was more prominent than that of fuel composition.

Figure 5 also shows BrC light-absorption properties reported in G-WISE-1.^44^ Similar to OA enhancement (Section 3.2), the BrC light-absorption properties in G-WISE-1 experiments clustered into three groups based on the combustion conditions (Wild, Rx, and WildDuff). Having a very low moisture content (∼2%),^44^ Wild emitted the darkest BrC across all G-WISE-1 and G-WISE-2 experimental permutations. Furthermore, Rx, which had similar moisture content to SF1 and SF2 and a mass loading (500 g/m^2^) between SF1 (750 g/m^2^) and SF2 (250 g/m^2^), emitted BrC with light-absorption properties falling between SF1 and SF2 (Figure 5). The BrC in WildDuff was substantially more absorbing than D1, which could be attributed to two factors. First, the duff samples in WildDuff and D1 were collected from different ecoregions and could have different characteristics (carbon content and compactness) that led to different combustion conditions. Second, both the surface fuels and duff in WildDuff had lower moisture content than in D1, leading to more efficient combustion.

Overall, results from both G-WISE-1 and G-WISE-2 are in line with the report of Saleh^33^ that high-temperature, more efficient combustion produces BrC with stronger light absorption than low-temperature, less-efficient combustion. The brown-black continuum proposed by Saleh et al.^78^ predicts that the increase in BrC light absorption is associated with an increase in molecular size. This relationship, which was supported by various studies,^35,126,127^ stems from the fact that as combustion temperature increases, organic precursors of BC undergo progressive molecular growth along the soot-formation pathway, producing larger and more strongly absorbing BrC molecules.^78^ Our findings, shown in Figure 6, further corroborate the brown-black continuum. The normalized unit-mass-resolution OA spectra revealed that the BrC produced in experimental permutations with more efficient combustion conditions was enriched in high *m/z* fragments, indicative of species with larger molecular sizes, compared to experimental permutations with less efficient combustion conditions. Notably, D1 and FD exhibited similar trends in OA mass spectra despite their different compositions, further supporting our assertions in the previous sections that the observed differences in physicochemical properties between SF1-SF5 and D1 were driven by differences in combustion conditions rather than fuel composition.

**Figure 6:**
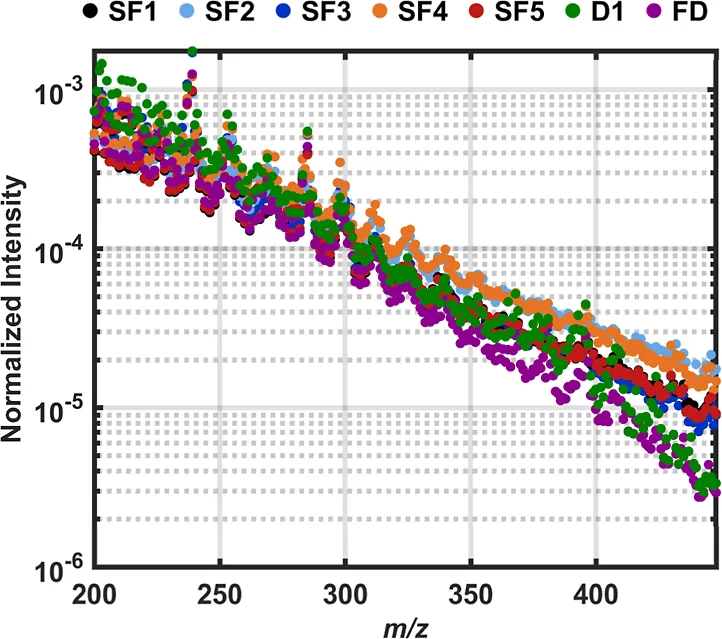
Unit-mass-resolution organic aerosol (OA) mass spectra normalized by total intensity measured by the SP-AMS.

## 4 Implications

There is a large variability in the reported extensive and intensive physicochemical properties of smoke emissions from wildland fires and laboratory burns of wildland fuels. Traditionally, the driving hypothesis for the underlying reason for this variability has been differences in fuel type, as manifested by laboratory study designs,^25,28,98,128–131^ field measurements,^132–135^ data compilations in review articles,^70,136,137^ and emission inventories^138–140^ being structured based on fuel type (or land-cover type). Differences in combustion conditions have been understood to play a role in this variability, as manifested in attempts to correlate emission properties with MCE^21,72,141–143^ and, to a lesser extent, BC/OA.^72,73^ However, differences in combustion conditions encountered between studies and within a study are usually incidental and have not been examined in a systematic fashion. Consequently, the perennial question of whether differences in fuel type or combustion conditions are more important in dictating smoke emissions has been largely unresolved, which was an impetus for G-WISE in general and this study in specific.

Here, we address this question using the proof-of-concept (FD) fuel bed, which included surface fuels only (SF) but had similar combustion conditions to the fuel bed that contained duff and surface fuels (D1) (Section 3.1). We show that the emissions from FD were much closer to D1 than SF in terms of (1) SOA formation potential (Section 3.2), (2) chemical composition of fresh and aged OA (Section 3.3), as well as (3) OA light-absorption properties (Section 3.4). These findings clearly demonstrate that combustion conditions were more important than fuel type in dictating the physicochemical properties of the OA emissions, at least the ones investigated in this study.

This conclusion can potentially be generalized across wildland fuels from other ecoregions. The reason is that the differences in molecular composition between the surface fuels and duff in this study are among the most profound when comparing wildland fuel types. Live and recently senesced wildland fuels (i.e., vegetation) have the same major dry components, which include cellulose, hemicellulose, lignin, extractives, and inorganic ash.^123,144^ The major difference in composition between live and recently senesced fuels is that live fuels have substantially higher water (moisture) content.^145^ The proportions of the dry components differ for the different parts of vegetation, where, for example, woody fuels often have higher cellulose and lignin contents but lower ash and extractives contents compared to fine fuels.^123,146^ These proportions also differ across vegetation types in different ecoregions, for example, between the Western U.S. (e.g., ponderosa pine, spruce) and Southeastern U.S. (e.g., long leaf pine, wire grass). The compositional difference between surface fuels and duff, in the same ecoregion, can be more drastic, as duff formation involves fundamental biochemical transformations, including microbial (fungal and bacterial) decomposition of cellulose and hemicellulose^101,103^ and, to a lesser extent, the fungal decomposition and mineralization of lignin.^101,103^ During these processes, preferential carbon consumption relative to nitrogen lowers the C/N of duff compared to undecomposed fuels.^101,103^ As shown in Figure S6, C/N of duff (45) was substantially lower than surface fuels (140) in our experiments, indicating a significant difference in composition between the two. This finding is consistent with previous studies generally reporting lower C/N of duff compared to live fuels and litter (surface fuels) for wildland fuels from various ecoregions across the U.S. and Canada.^143,147^ Furthermore, the compositional differences (C/N) between surface fuels and duff in this study are among the largest when comparing the wide variety of fuel types across ecoregions in the U.S. and Canada (Figure S6). Therefore, the fact that the conclusion in this study (combustion conditions are more important than fuel type) was obtained from comparing duff and surface fuels suggests that it applies to other fuel types that have less drastic differences in molecular composition.

A practical implication is that combustion conditions (e.g., MCE, BC/OA, other metrics, or a combination of metrics) can constitute a unifying framework for accounting for the variability in physicochemical properties in emissions from wildland fires, regardless of vegetation (land-cover) type. We note that this assertion does not imply that the detailed molecular composition of the emissions is solely dictated by combustion conditions, as the presence of certain molecules in the emissions surely depends on the molecular composition of the fuel, i.e., fuel type.^147,148^ Rather, combustion conditions dictate the physicochemical properties that are governed by a broad range of smoke constituents, such as those investigated in this study: the relative abundance of chemical families, light-absorption properties, and SOA formation potential.

We also note that combustion conditions and fuel type are not independent. As we show here and in previous studies,^29,44,47^ combustion conditions emerge from the complex interactions between fuel type (or, more accurately, fuel-bed composition), other fuel-bed characteristics (e.g., dry mass loading), and environmental conditions (e.g., manifested as moisture content). Furthermore, fuel availability (i.e., whether the fuel ignites or not) and, consequently, combustion conditions are largely dependent on environmental conditions. For the same land-cover type, only surface fuels would be available for combustion under moderate moisture-content conditions, but duff and large woody fuels can become available during drought conditions, leading to significant differences in combustion conditions and, consequently, smoke properties between the two scenarios.

## Supplementary Material



## Notes

The Supporting Information includes information on OH exposure and OA enhancement calculations, measurement uncertainty and statistical analyses, supplementary SP-AMS mass spectra, the G-WISE-2 experimental matrix, a Van Krevelen diagram, additional OA marker analyses (*f*_73_-*f*_60_ relationships), equivalent photochemical ages, carbon-to-nitrogen (C/N) ratios of the fuels used in this study and comparison with literature values, fuel consumption data, and tables containing the numerical values and associated standard deviations used to generate Figures 1, 2, 4 and 5 (PDF)
