# Supplementary material for: Combustion Conditions are the Major Drivers of the Variability in Organic Aerosol Chemical Composition and Secondary Organic Aerosol Formation in Emissions from Simulated Wildland Fires
Source: ACS EST Air. 2026 Sep 15;3(10):2843–58. doi: 10.1021/acsestair.6c00258 (PMC13660632; doi:10.1021/acsestair.6c00258)
Supplement: Supplementary file 1 [file ea6c00258_si_001.pdf]

Supporting information for:

Combustion Conditions are the Major Drivers of the Variability in Organic Aerosol Chemical Composition and Secondary Organic Aerosol Formation in Emissions from Simulated Wildland Fires

Muhammad I. Abdurrahman[1], Michael T. Caraway[1], Nicholas Kusumo[1], Thomas Carroll[1,2], Kruthika V. Kumar[1], Anita Anosike[1], Ariana M. Deegan[3], Ryan P. Poland[3], John T. Allen[3], Mac A. Callaham[4], Joseph J. O'Brien[4], Edward C. Fortner[5], Amanda Frossard[3], Geoffrey D. Smith[3], Rawad Saleh[1]*

[1]School of Environmental, Civil, Agricultural, and Mechanical Engineering, University of Georgia, Athens, GA, 30602, USA.

[2]Department of Physics and Astronomy, University of Georgia, Athens, Georgia 30602, United States.

[3]Department of Chemistry, University of Georgia, Athens, GA 30602, USA.

[4]U.S. Department of Agriculture Forest Service, Southern Research Station, Athens Prescribed Fire Science Laboratory, Athens, Georgia, USA.

[5]Aerodyne Research Inc., Billerica, Massachusetts, USA.

*Corresponding author

S1. OH Exposure in the Oxidation Flow Reactor

OH exposure in the oxidation flow reactor (OFR) was estimated from toluene consumption, assuming toluene consumption is dominated by a pseudo-first-order reaction with OH[1]:

$$\mathrm{OH_{exp}} = -\frac{1}{k_{\mathrm{OH+Tol}}}\ln\left(\frac{[\mathrm{Tol}]_{\mathrm{final}}}{[\mathrm{Tol}]_{\mathrm{initial}}}\right) \qquad \text{(S1)}$$

Where $k_{OH} = 6.11 \times 10^{-12}$ cm^3 molecule^{-1}s^{-1} is the rate constant of toluene reaction with OH at 298 K.[2] $[\mathrm{Tol}]_{\mathrm{initial}}$ is the initial concentration of toluene downstream of the OFR when the UV lights are off. $[\mathrm{Tol}]_{\mathrm{final}}$ is the concentration of toluene measured using the AROMAVOC downstream of the OFR when the OFR UV lights are turned on. The OH exposure was converted to an equivalent photochemical age (days) using:

$$\text{Photochemical age (days)} = \frac{\mathrm{OH_{exp}}}{[\mathrm{OH}]_{\mathrm{amb}} \times 86400} \qquad \text{(S2)}$$

Where, $[\mathrm{OH}]_{\mathrm{amb}} = 1.5 \times 10^6$ molecules cm^{-3} is the average OH concentration in the atmosphere.[3,4]

S2. Organic Aerosol Enhancement

The estimation of organic aerosol (OA) enhancement is illustrated in Figure S7, which presents a time series of OA mass concentration for a representative experiment, measured using an Aerodyne soot-particle aerosol mass spectrometer (SP-AMS). As described in Section 2.4 in the main text, the SP-AMS was alternated between laser-ON and laser-OFF modes. In this study, we focus on measurements obtained during laser-on operation, which captures both refractory and non-

refractory OA. At the beginning of each experiment, fresh aerosols were sampled for approximately 30 minutes, during which the UV lights in the OFR were kept off, and the measured OA mass concentration represents the fresh OA. When the UV lights in the OFR were turned on, the OA concentration increased relative to the fresh OA baseline, indicating the formation of SOA from the photochemical oxidation of gas-phase precursors. The OA concentration, however, decreased over time due to particle-wall losses and infiltration-induced dilution. Therefore, to quantify the OA enhancement, we fitted an exponential decay function to the fresh concentration (measured during the UV light-OFF period), yielding estimated concentrations of fresh OA that would have been measured in the absence of photochemical aging at the corresponding measurement times. The difference between the fitted fresh OA baseline and the measured OA concentration (OFR UV lights-ON) was used to calculate the net increase in OA mass concentration resulting from formation of secondary organic aerosol (SOA), i.e., OA enhancement.

S3. Measurement Uncertainty and Calculation of Standard Deviations

Measurement uncertainty was evaluated based on the time resolution of the corresponding measurement and the nature of the calculation used to obtain the reported value. For all data, the values shown in Figures 1, 2, 4, and 5 in the main text represent the mean value for each experimental permutation, and the uncertainty bars are not shown because they were minimal, and in most cases, they did not exceed the marker size, but are listed in Tables S3, S4, and S6.

The standard deviation was calculated as:

$$\sigma = \sqrt{\frac{\sum_{i=1}^{n}(x_i - \bar{x})^2}{n-1}} \qquad \text{(S3)}$$

Where x_i is an individual measurement, $\bar{x}$ is the mean of the measurements, and n is the number of measurements used in the calculation. The mean was calculated as:

$$\bar{x} = \frac{1}{n}\sum_{i=1}^{n} x_i \qquad \text{(S4)}$$

Below, we describe the approaches used to determine the uncertainty associated with the data obtained from different measurements/instruments.

S3.1 Uncertainty in Modified Combustion Efficiency (MCE)

MCE was calculated from the CO and CO_2 measurements using Equation 1 in the main text. CO and CO_2 were both measured at a time resolution of 1 minute. Therefore, an MCE value was calculated for each minute during the one-hour fresh-emission sampling period. The mean MCE values reported in Figure 1 for each experimental permutation were calculated from these one-minute resolution values, and the corresponding uncertainty values were calculated as standard deviations of these MCE values using Equation S3. The standard deviations in MCE were small relative to the differences observed among the experimental permutations. The mean MCE values and their corresponding standard deviations are provided in Table S3.

S3.2 Uncertainty in Black carbon to organic aerosol ratio (BC/OA)

BC/OA was determined using two methods, i.e., measurements from (1) the organic carbon elemental carbon (OCEC) analyzer and (2) the SP-AMS. For the OCEC measurements, the uncertainty associated with the reported OC and EC concentrations was retrieved directly from the uncertainty information provided by the instrument, which reports uncertainty associated with the thermal-optical determination of OC and EC for each filter sample. The uncertainty in BC/OA was then calculated by propagating the uncertainties in BC and OA according to:

$$\sigma_{BC/OA} = \frac{BC}{OA}\sqrt{\left(\frac{\sigma_{BC}}{BC}\right)^2 + \left(\frac{\sigma_{OA}}{OA}\right)^2} \qquad (S5)$$

where σ_{BC} and σ_{OA} are the uncertainties associated with the BC and OA measurements, respectively.

For the SP-AMS, measurements of both BC and OA were obtained at a time resolution of 1 min throughout the duration of the fresh-emission sampling period. The mean BC/OA from the SP-AMS, which was reported in Figure 1, for each experimental permutation was calculated using Equation S4, and the uncertainty, which was reported as a standard deviation, was calculated using Equation S3. As we noted in the main text, the resulting standard deviations of both MCE and BC/OA were generally smaller than the markers that were used in the figures. The mean values and corresponding standard deviations are also provided in Table S3.

S3.3 Uncertainty in Organic Aerosol Enhancement

OA enhancement was calculated using the difference between the measured OA concentration during photochemical aging and the fresh OA baseline, as described in Section 2.4 of the main text and Section S2. OA enhancement was calculated at one-minute resolution using the OA concentrations retrieved from the SP-AMS. The mean OA enhancement reported in Figure 2 for each experimental permutation was calculated from the individual enhancement values using Equation S4, and the uncertainty for each experimental permutation was calculated using Equation S1as a standard deviation of the enhancement values. The mean OA enhancement values and their corresponding standard deviations are provided in Table S3.

S3.4 Uncertainty in the SP-AMS OA Markers

The SP-AMS characteristic markers of OA, f_{44}, f_{43}, and f_{60}, were retrieved from the SP-AMS at one-minute resolution. The mean values of each marker were calculated separately for the fresh and aged sampling periods using Equation S4. The corresponding uncertainties were calculated as standard deviations of the time-resolved measurements within each sampling period using Equation S3. The mean values for each marker and their corresponding standard deviations are provided in Table S4.

S3.5 Uncertainty in Light-Absorption Properties

The imaginary part of the OA refractive index at 550 nm k_{550}, and its wavelength dependence, w, were retrieved using the optical-closure approach described in Section 2.6 of the main text. The method takes, as inputs, the aerosol size distribution measured by the SMPS and the absorption coefficient measured by the photoacoustic spectrometer. The SMPS size distribution was measured at a time resolution of approximately 90 seconds, whereas the absorption coefficient was measured at a time resolution of 1 second. To match the two measurements, the absorption measurements were therefore averaged at the corresponding SMPS time stamps before the optical-closure calculations were performed. For each experimental permutation, five paired SMPS and absorption measurements, each having a 90-seconds time resolution, were selected from the fresh sampling period. Therefore, the optical closure analysis yielded five sets of k_{550} and w values for each experimental permutation, from which means and standard deviations were calculated using Equation S4 and Equation S3, respectively. The mean values of k_{550} and w and their corresponding standard deviations are provided in Table S6.

Tables

Table S1. G-WISE-2 experimental matrix

Experimental Permutation	Isolated variable	Surface fuel dry mass loading (g m^{-2})	Fine fuels moisture content	Duff dry mass loading (g m^{-2})	Duff moisture content
SF1	baseline	750	12%	N/A	N/A
SF2	Surface fuels mass loading	250	12%	N/A	N/A
SF3	Surface fuels mass loading	1500	12%	N/A	N/A
SF4	Surface fuels moisture content	750	5%	N/A	N/A
SF5	Surface fuels moisture content	750	18%	N/A	N/A
SF6[1]	Fuel bed area (1 m^2)	750	12%	N/A	N/A
D1	Include duff (duff moisture content)	750	12%	4307 - 6414	5%
D2	Include duff (duff moisture content)	750	12%	4822 - 5840	20%
D3	Include duff (duff moisture content)	750	12%	5059 - 6551	60%
D4[2]	Include duff (wind speed = 0.5 m/s)	750	12%	5902	5%
FD[3]	Faux Duff	1500	30%	N/A	N/A
DD[4]	Decompacted Duff	170	12%	1400	5%

[1]Fuel bed area for all other permutations is 0.5 m^2
[2]Wind speed for all other permutations is 1 m/s
[3]See main text for description of FD
[4]Duff was manually decompacted to test the relative importance of compactness versus low carbon content in the low-efficiency combustion conditions exhibited by duff.

Table S2. OH exposures calculated from toluene consumption and the corresponding equivalent atmospheric ages.

Experimental Permutation	OH exposure (molec. $cm^{-3}\cdot s$)	Photochemical age (days)
SF1	5.59E+11	4.31
SF2	N/A	N/A
SF3	6.27E+11	4.84
SF4	5.72E+11	4.41
SF5	5.46E+11	4.22
D1	5.68E+11	4.38
FD	7.26E+11	5.60

Table S3. Numerical values (mean ± standard deviation) of data points in Figure 1 and Figure 2 of the main text

Experimental Permutation	MCE	BC/OA (OCEC Analyzer)	BC/OA SP-AMS (Total)	BC/OA SP-AMS (C1-C4)	OA Enhancement (%)
SF1	0.924 ± 0.001	0.030 ± 0.001	0.0386± 0.0010	0.0368 ± 0.0010	31.34 ± 0.9
SF2	0.942 ± 0.002	0.065 ± 0.002	0.0683 ± 0.0015	0.0541 ± 0.0012	25.90 ± 2.1
SF3	0.907 ± 0.002	0.025 ± 0.001	0.0258 ± 0.0008	0.0179 ± 0.0007	37.10 ± 1.0
SF4	0.932 ± 0.001	0.072 ± 0.002	0.0726 ± 0.0015	0.0565 ± 0.0012	15.80 ± 1.0
SF5	0.923 ± 0.001	0.028 ± 0.001	0.0250 ± 0.0008	0.0230 ± 0.0008	37.34 ± 1.2
D1	0.776 ± 0.003	0.007 ± 0.001	0.1211 ± 0.0020	0.0095 ± 0.0008	87.49 ± 5.3

FD	0.791 ± 0.002	0.008 ± 0.001	0.1434 ± 0.0025	0.0147 ± 0.0009	69.64 ± 3.1

Table S4. Numerical values (mean ± standard deviation) for data points shown in Figure 4 of the main text

Experimental Permutation	f_{43} Fresh	f_{43} Aged	f_{44} Fresh	f_{44} Aged	f_{60} Fresh	f_{60} Aged
SF1	0.0599 ± 0.0008	0.0691 ± 0.0005	0.0405 ± 0.0013	0.0468 ± 0.0007	0.0352 ± 0.0014	0.0385 ± 0.0005
SF2	0.0515 ± 0.0012	0.0567 ± 0.0014	0.0440 ± 0.0017	0.0646 ± 0.0020	0.0258 ± 0.0006	0.0236 ± 0.0007
SF3	0.0536 ± 0.0006	0.0618 ± 0.0004	0.0358 ± 0.0012	0.0445 ± 0.0004	0.0299 ± 0.0014	0.0324 ± 0.0004
SF4	0.0538 ± 0.0005	0.0602 ± 0.0006	0.0435 ± 0.0006	0.0629 ± 0.0008	0.0415 ± 0.0007	0.0352 ± 0.0004
SF5	0.0622 ± 0.0006	0.0707 ± 0.0005	0.0356 ± 0.0007	0.0458 ± 0.0012	0.0404 ± 0.0008	0.0415 ± 0.0006
D1	0.0694 ± 0.0002	0.0747 ± 0.0002	0.0195 ± 0.0002	0.0274 ± 0.0004	0.0333 ± 0.0003	0.0328 ± 0.0003
FD	0.0683 ± 0.0002	0.0769 ± 0.0002	0.0263 ± 0.0003	0.0352 ± 0.0004	0.0449 ± 0.0004	0.0448 ± 0.0004

Table S5. Consumption of woody fuels

Experimental Permutation	Consumption Fraction
SF1	8.23 %
SF2	9.89 %
SF3	13.45 %
SF4	47.41 %
SF5	10.28 %

Table S6. Numerical values (mean ± standard deviation) of data points in Figure 5 of the main text.

Experimental Permutation	k_{550}	w
SF1	0.0069 ± 0.0004	3.48 ± 0.81
SF2	0.0195 ± 0.0042	1.47 ± 0.48
SF3	0.0040 ± 0.0001	4.42 ± 0.58
SF4	0.0181 ± 0.0041	1.97 ± 0.20
SF5	0.0037 ± 0.0009	4.81 ± 0.16
D1	0.0013 ± 0.0001	6.99 ± 0.30
FD	0.0017 ± 0.0001	6.38 ± 0.54

Figures

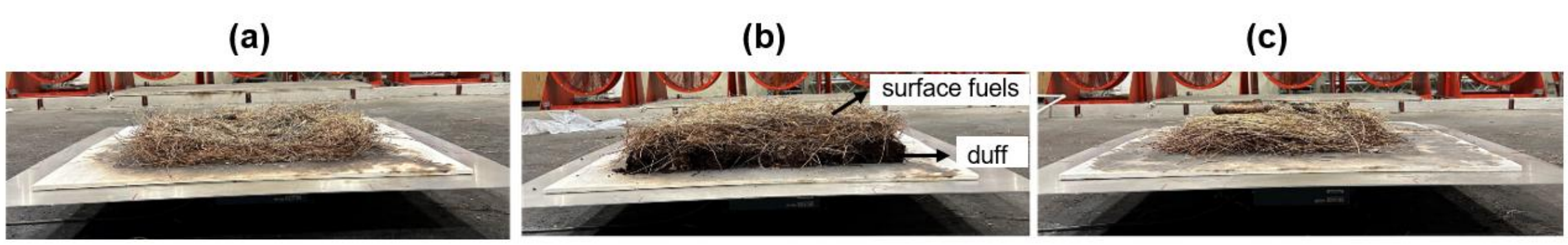

Figure S1. Representative side-view photographs of the fuel-bed configurations used in this study. (a) SF1 consisted of surface fuels only with a dry mass loading of 750 g m^{-2}. (b) D1 consisted of

surface fuels with the same mass loading as SF1 on top of a duff layer with a dry mass loading of 5844 g m^{-2}. (c) FD consisted of surface fuels only with a dry mass loading of 1500 g m^{-2} that were compacted and moistened to reproduce similar inefficient combustion conditions to D1.

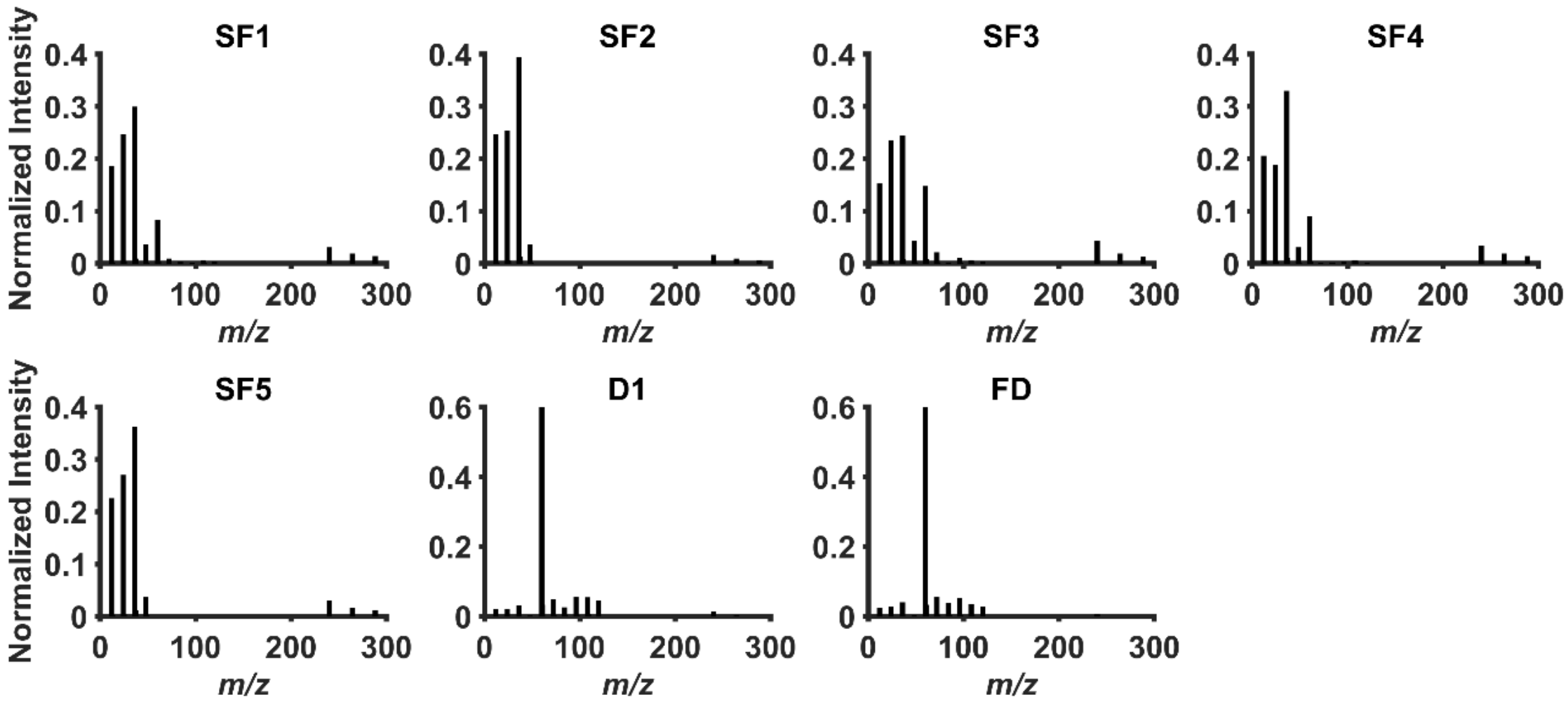

Figure S2. Normalized refractory black carbon (rBC) mass spectra measured by the SP-AMS.

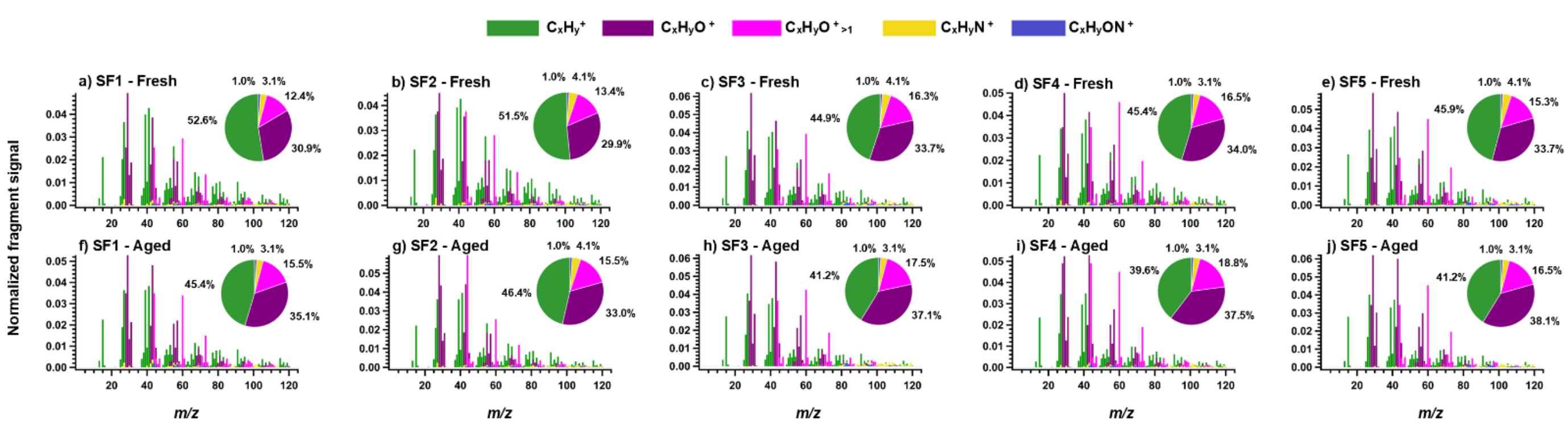

Figure S3. Normalized high-resolution mass spectra of organic fragment ions and relative abundance of chemical families in the organic aerosol (OA) measured by the SP-AMS for experimental permutations involving only surface fuels (SF1-SF5). (a-e) Fresh OA mass spectra, and (f-j) aged OA mass spectra.

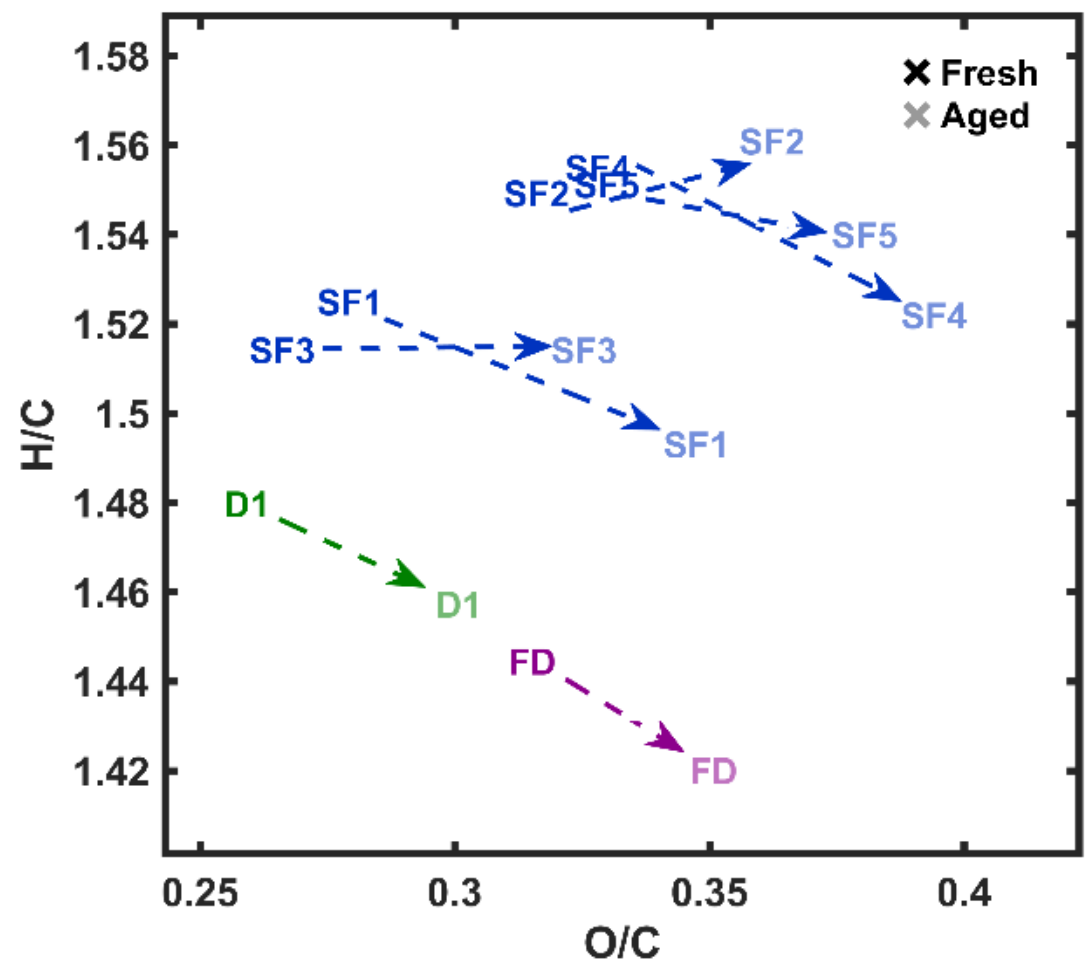

Figure S4. Van Krevelen diagram showing the hydrogen-to-carbon (H/C) versus oxygen-to-carbon (O/C) atomic ratios of the organic aerosol (OA) obtained using the SP-AMS. Bold identifiers denote fresh OA (OFR lights-OFF), and faded identifiers denote aged OA (OFR lights-ON), with arrows indicating the direction of photochemical aging.

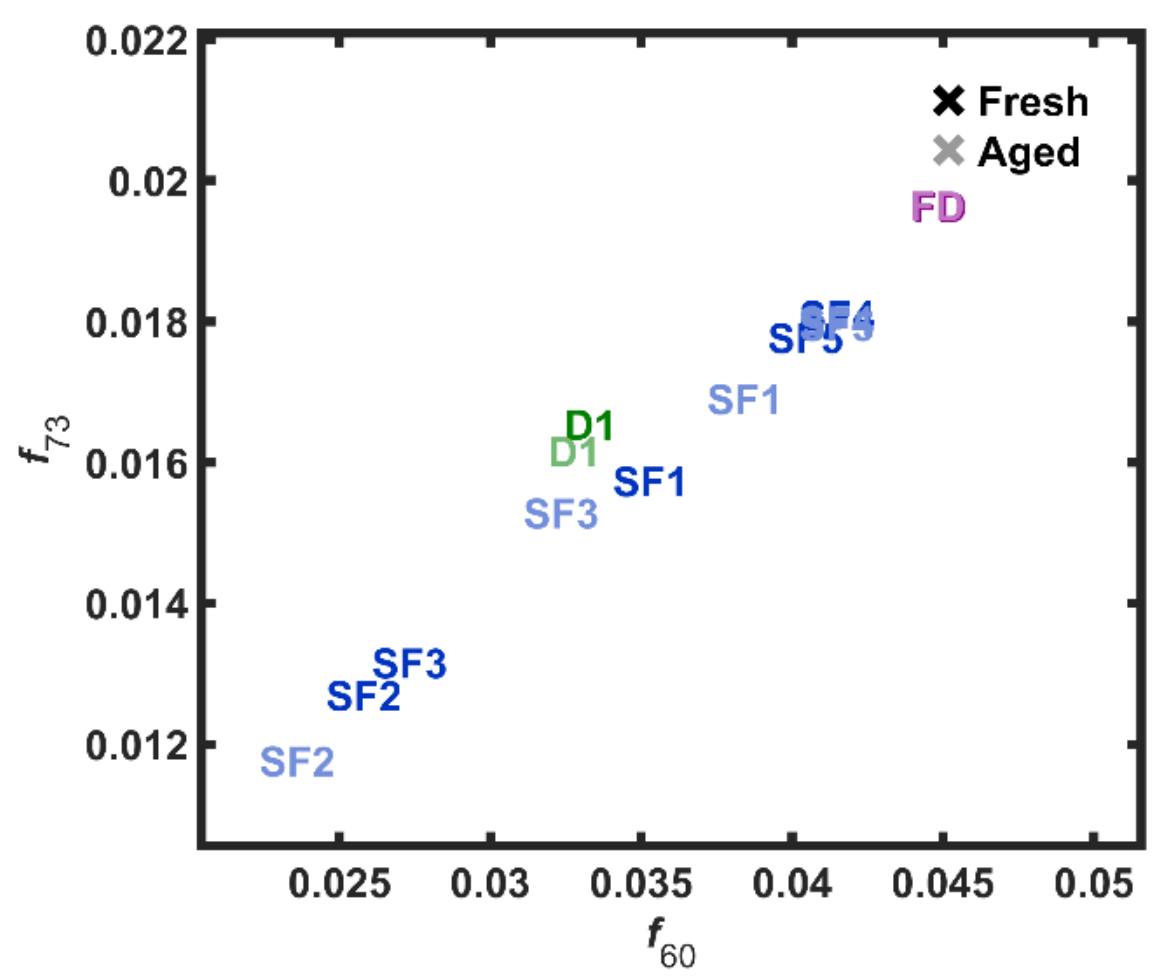

Figure S5. Organic markers for fresh and aged organic aerosol (OA) measured by the SP-AMS and presented in f_{73} versus f_{60} space. Bold identifiers denote fresh OA (OFR UV lights-OFF) and

faded identifiers denote aged OA (OFR UV lights-ON)

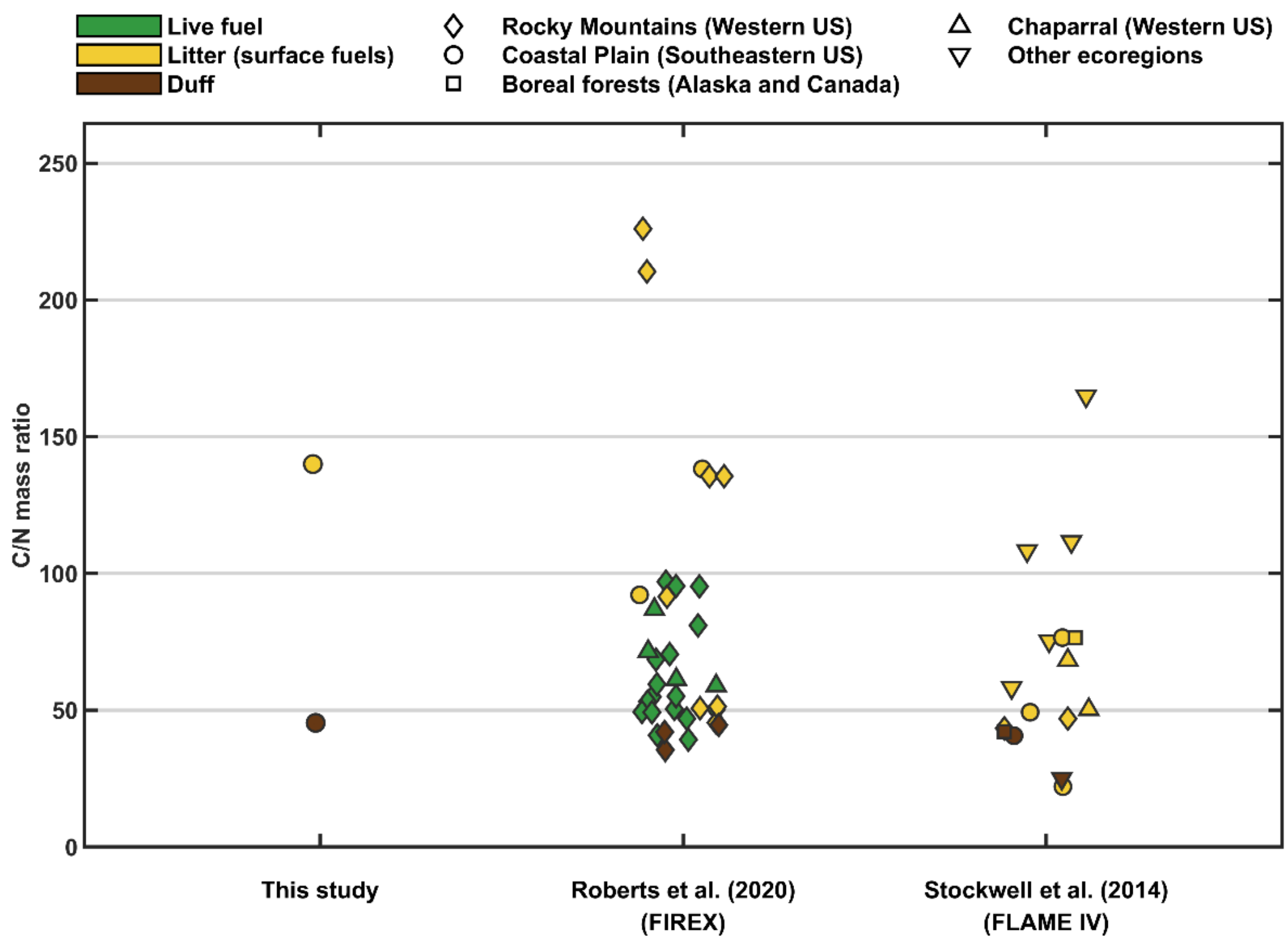

Figure S6. Carbon-to-nitrogen (C/N) mass ratios of wildland fuels in this study and reported by previous studies: Roberts et al.[5] and Stockwell et al.[6]

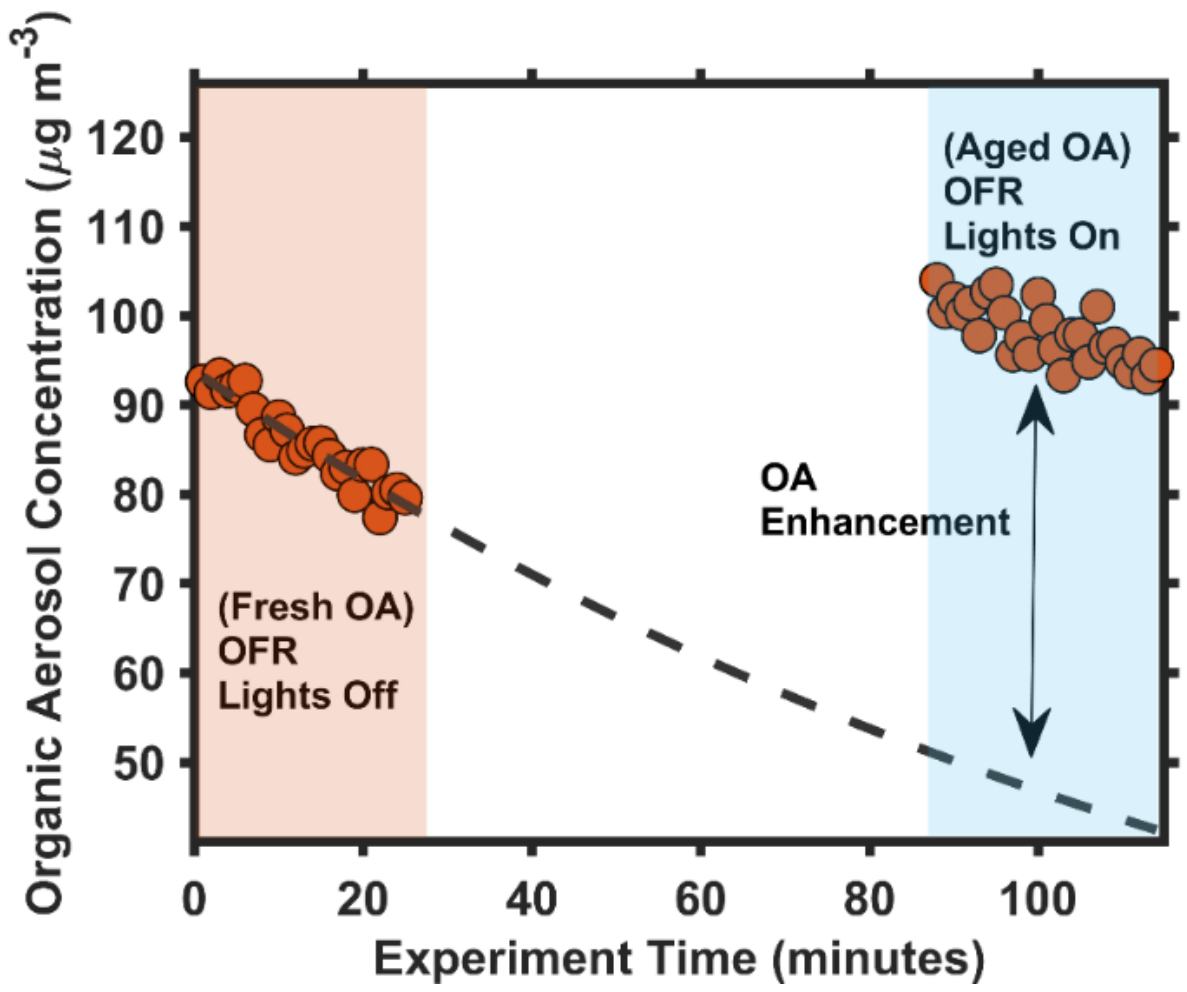

Figure S7. Time series of organic aerosol (OA) mass concentration illustrating calculation of OA enhancement for a representative experiment.

References

(1) Srivastava, D.; Li, W.; Tong, S.; Shi, Z.; Harrison, R. M. Characterization of Products Formed from the Oxidation of Toluene and *m*-Xylene with Varying NOx and OH Exposure. *Chemosphere* **2023**, *334*, 139002. https://doi.org/10.1016/j.chemosphere.2023.139002.

(2) Davis, D. D.; Bollinger, W.; Fischer, S. Kinetics Study of the Reaction of the Hydroxyl Free Radical with Aromatic Compounds. I. Absolute Rate Constants for Reaction with Benzene and Toluene at 300.Deg.K. *J. Phys. Chem.* **1975**, *79* (3), 293–294. https://doi.org/10.1021/j100570a021.

(3) Mao, J.; Ren, X.; Brune, W. H.; Olson, J. R.; Crawford, J. H.; Fried, A.; Huey, L. G.; Cohen, R. C.; Heikes, B.; Singh, H. B.; Blake, D. R.; Sachse, G. W.; Diskin, G. S.; Hall, S. R.; Shetter, R. E. Airborne Measurement of OH Reactivity during INTEX-B. *Atmos. Chem. Phys.* **2009**, *9* (1), 163–173. https://doi.org/10.5194/acp-9-163-2009.

(4) Palm, B. B.; Peng, Q.; Fredrickson, C. D.; Lee, B. H.; Garofalo, L. A.; Pothier, M. A.; Kreidenweis, S. M.; Farmer, D. K.; Pokhrel, R. P.; Shen, Y.; Murphy, S. M.; Permar, W.; Hu, L.; Campos, T. L.; Hall, S. R.; Ullmann, K.; Zhang, X.; Flocke, F.; Fischer, E. V.; Thornton, J. A. Quantification of Organic Aerosol and Brown Carbon Evolution in Fresh Wildfire Plumes. *Proc. Natl. Acad. Sci.* **2020**, *117* (47), 29469–29477. https://doi.org/10.1073/pnas.2012218117.

(5) Roberts, J. M.; Stockwell, C. E.; Yokelson, R. J.; de Gouw, J.; Liu, Y.; Selimovic, V.; Koss, A. R.; Sekimoto, K.; Coggon, M. M.; Yuan, B.; Zarzana, K. J.; Brown, S. S.; Santin, C.; Doerr, S. H.; Warneke, C. The Nitrogen Budget of Laboratory-Simulated Western US Wildfires during the FIREX 2016 Fire Lab Study. *Atmos. Chem. Phys.* **2020**, *20* (14), 8807–8826. https://doi.org/10.5194/acp-20-8807-2020.

(6) Stockwell, C. E.; Yokelson, R. J.; Kreidenweis, S. M.; Robinson, A. L.; DeMott, P. J.; Sullivan, R. C.; Reardon, J.; Ryan, K. C.; Griffith, D. W. T.; Stevens, L. Trace Gas Emissions from Combustion of Peat, Crop Residue, Domestic Biofuels, Grasses, and Other Fuels: Configuration and Fourier Transform Infrared (FTIR) Component of the Fourth Fire Lab at Missoula Experiment (FLAME-4). *Atmos. Chem. Phys.* **2014**, *14* (18), 9727–9754. https://doi.org/10.5194/acp-14-9727-2014.
